# Reverse Engineering the Cooperative Machinery of Human Hemoglobin

**DOI:** 10.1371/journal.pone.0077363

**Published:** 2013-11-27

**Authors:** Zhong Ren

**Affiliations:** 1 Center for Advanced Radiation Sources, The University of Chicago, Argonne, Illinois, United States of America; 2 Renz Research, Inc., Westmont, Illinois, United States of America; University of South Florida College of Medicine, United States of America

## Abstract

Hemoglobin transports molecular oxygen from the lungs to all human tissues for cellular respiration. Its α_2_β_2_ tetrameric assembly undergoes cooperative binding and releasing of oxygen for superior efficiency and responsiveness. Over past decades, hundreds of hemoglobin structures were determined under a wide range of conditions for investigation of molecular mechanism of cooperativity. Based on a joint analysis of hemoglobin structures in the Protein Data Bank (Ren, companion article), here I present a reverse engineering approach to elucidate how two subunits within each dimer reciprocate identical motions that achieves intradimer cooperativity, how ligand-induced structural signals from two subunits are integrated to drive quaternary rotation, and how the structural environment at the oxygen binding sites alter their binding affinity. This mechanical model reveals the intricate design that achieves the cooperative mechanism and has previously been masked by inconsistent structural fluctuations. A number of competing theories on hemoglobin cooperativity and broader protein allostery are reconciled and unified.

## Introduction

A variety of quaternary assemblies [1], [2] of hemoglobins (Hb) suggests diverse mechanisms of cooperativity. However, the current understanding of Hb structure-function relationship does not explain the diverse molecular strategies. The sequential model by Pauling-KNF [3] and the symmetry model by MWC [4] are the symbols of two major camps in a prolonged debate for decades.

Human and other tetrameric Hbs undergo large quaternary change between the low affinity, unligated T state and high affinity, ligated R state, with structural motions greater than 10 Å at one point of the tetrameric structure. This large structural change originates from a small shift of Fe position in the heme groups of only a few 10^th^ of Å upon change in ligation state. How does a small motion amplify and propagate through protein? What is the structural basis of oxygen binding affinity, and how is it modulated by the large quaternary change? These fundamental questions regarding protein structure-function relationship have been asked, answered, and debated repeatedly for decades. In this work, I present not a single new structure, but employ a reverse engineering approach to an overall interpretation of structural dynamics and functional mechanism based on hundreds of known Hb structures to date. The companion article [5] describes a large-scale structural meta-analysis that provides a global perspective of the entire collection of tetrameric Hb structures and guides this reverse engineering approach away from inconsistent structural fluctuations. My analysis presented in these two companion articles reconciles seemingly conflicting aspects and unites major theories on allosteric regulation. See Materials and Methods (MM) of the companion article for a list of notations used in both articles. The relationship among the diverse topics presented in this and the companion articles is outlined in MM below, which may serve as a guide to readers.

**Figure 1:**
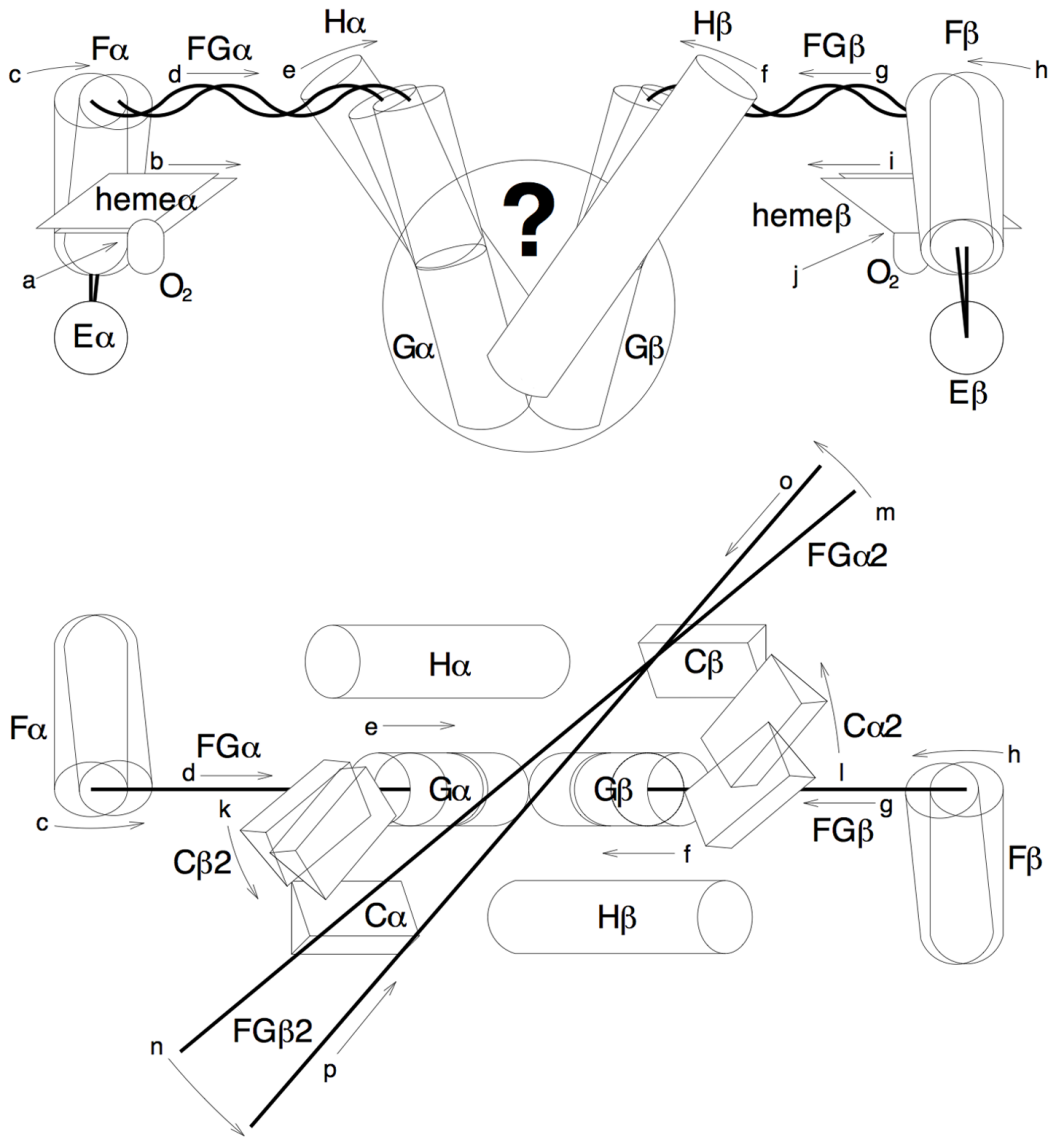
Summary of cooperative actions. α and 3/10 helices are schematically represented by cylinders and triangular columns, respectively. Loops are represented by thick lines. Arrows are alphabetically labeled and indicate motions in order. The top panel shows one dimer with the dimer interface facing up. Compare to a similar view of the experimental structure in Movie S1. Some components are stretched out for clarity. The bottom panel shows the same dimer viewed from the opposite dimer. The two crossing thick lines in the bottom panel mark the directions of FGs of the second dimer before and after the quaternary rotation, but Gs are omitted for clarity. Compare to a similar view of the experimental structure in Movie S2. Hb structure as-is in the unligated T state (also known as species 01 according to the microstates theory [6], [17]) is not suited for oxygen binding, because each ligand binding site is preoccupied by a couple of distal residues in E (Fig. S2), resulting in reduced affinity compared to a free monomeric globin [54]. The distal Es are very rigid and do not move upon oxygen binding. To clear enough space for a primary oxygen binding event in one subunit (a), the proximal F (c), to which the heme group (b) anchors, moves towards the center of the molecule to avoid the distal residues in E (species 11 or 12; Trttt or Ttrtt in tertiary two-state notation [7]). This results in an inward motion of FG (d) that permits straightening of a pre-bent G in T state (e). This motion of stereochemical avoidance induced by a primary oxygen binding is reciprocated mechanically in the partner subunit via a lever system of helices at the αβ dimeric core (big ?) that produces identical motions in the partner subunit (f, g, and h). As the oxygen binding site in the partner subunit clears (i), a second oxygen binds (j) in the same dimer (species 21 or Trrtt). As a result of these intradimer cooperative bindings, a large pinch motion brings two FGs closer to each other in the first ligated dimer (d and g). The forces of pinch are applied on two Cs that firmly hold a constant distance between them in the second dimer where no oxygen binding takes place yet (species 21 or Trrtt). To resolve this conflict, two dimers rotate with respect to each other and convert to R state (k, l, m, and n). This quaternary rotation also brings two FGs in the second deoxy dimer closer, due to direct mechanical couplings between two dimers (o and p). Oxygen binding affinity increases in the second deoxy dimer because the spaces at these binding sites are cleared up consequently. Similar to the intradimer cooperative events in the first dimer, the first oxygen binding in the second dimer (species 31 or 32; Rrrrt or Rrrtr) further promotes oxygen binding at the last binding site. Finally, four oxygen bindings (species 41 or Rrrrr) occur with ever increasing affinity. This sequence of events explains the mechanism of cooperative oxygen binding by tetrameric Hb.

The main findings of this study are summarized in a strip of cartoons (Fig. 1) showing the cooperative model of human Hb in action. This structural mechanism explicitly illustrates (as Pauling-KNF sequential model hypothesizes) the sequence of molecular events and the inner workings of the cooperative machinery that undergoes spontaneous symmetry-preserving interconversion between the two discrete quaternary states of MWC. This model supports both the molecular code of Ackers et al. [6] and the tertiary two-state theory of Eaton and coworkers [7], [8], and explains why both are in complete agreement with other theories. The molecular code [6] is not only correct, but also the logical proof of symmetry conservation, the centerpiece of MWC. The tertiary two-state theory [7], [8] is necessary to model time-resolved data. The companion article further develops this analytical technique to hypothesize asymmetric structures of transient intermediates that communicate signals of ligation states between subunits and dimers, although these short-lived structural species have not been observed in the collection of static structures [5]. I shall present evidences gathered from hundreds of Hb structures in PDB to support my theory (Fig. 1) and to provide structural foundation for other theories on allosteric regulation.

## Results

### Invariant frameworks in the tetrameric architecture

The tetrameric architecture of Hb is unique in the sense that the molecule is hollow at its center with two well separate structural cores located at two opposite poles of the molecule. The overall molecular shape changes upon different quaternary states [9] (Fig. S1). The central cavity shrinks slightly in R state compared to that in T state. At each core, Bs, Gs, and Hs from α and β form a tight α–β interface [10]. In contrast, two dimers are assembled into a tetramer via only four small couplings between FGs and Cs from different chains, that is, FGαi-Cβi and FGβi-Cαi (see MM of the companion article for notation). The surface of the tetramer is largely covered by As, Es, and Fs (including F′s) from the four subunits. Each E–F pair sandwiches a heme group on the surface of the molecule (Fig. S1) so that every oxygen binding site is equally accessible when the exact space of the ligand is vacant (Fig. S2). This unique architecture hints that the interplay between two dimers plays a key role in Hb function.

**Figure 2:**
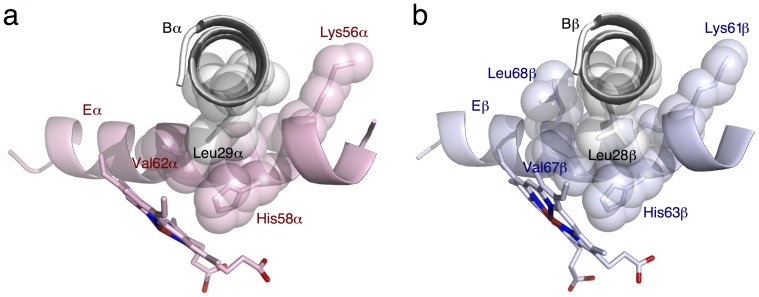
Interhelix B–E junctions. **a**. α. **b**. β. Gly59α 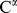 is 3.5 Å from the peptide plane of Gly25α-Ala26α, so is Gly64β 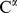 from the peptide plane of Gly24β-Gly25β.

Using a new computational algorithm based on distance matrix that involves no structural alignment (MM) [5], the invariant structural framework of a dimer is identified from hundreds of dimer structures. It largely coincides with α–β interface, but further extends to parts of Cs and Es on the surface of the molecule (Figs. S3 and S4). The unusually tight junction between two helices we found in dimeric Hbs from invertebrates [11] also occurs here in human Hb, although at different locations. Parameterization of helices (Eq. 1; MM) shows that B and E in human Hb cross each other at a very close axis-to-axis distance of about 6.6 Å (Fig. S5). At the point of crossing, Gly22α, Gly25α, and Ala26α are on Bα, and Gly59α at E8 is on Eα. These small residues allow direct backbone contacts between the crossing helices. The equivalent residues in β resulting in backbone contacts are Gly24β, Gly25β, and Gly64β at E8 (Fig. 2). It is noteworthy that residues flanking the crossing point often carry large side chains from both helices, such as Leu29α, Lys56α, Lys60α, Leu28β, Lys61β, Lys65β, and Leu68β. The combination of these small and large residues forms a dovetail structure that interlocks two crossing helices. More importantly, each tight interhelix junction at E8 Gly is strategically located right next to the distal His at E7 and is one helical turn away from the distal Val at E11 (Fig. 2). This B–E dovetail structure firmly holds the N-terminal section of E to the dimeric core, where B is an important member (Figs. S3 and S4), and thus extends the invariant framework from the core to the molecular surface. Similarly, another close interhelix junction that consists of small residues occurs at the ends of Gα and Gβ (Fig. S6), which is one of three major parts of α–β interface.

It was immediately obvious to Perutz [12] and many others from the deoxy conformation around the heme groups in T state that any ligand binding in α or β requires substantial relocations of distal His58α and Val62α or distal His63β and especially Val67β in the N-terminal parts of Es, because these residues occupy the ligand spaces (Fig. S2). However, these short sections of Es that suppose to be mobile enough to allow relocations of these distal residues are also included in the most immobile framework because they are firmly held to the dimeric cores by the dovetailed helix crossings. Such apparent contradiction suggests great functional significance of the immobile N-termini of Es. I shall demonstrate that these intra- and inter-subunit backbone contacts are important to the function.

### Rigid distal block and mobile allosteric core

The stereochemical hindrance at the oxygen binding sites in T state is resolved not by moving the distal His and Val, since this section of E is reinforced by a special design of interhelix junction (Fig. 2). Merely moving the side chains is not nearly enough to resolve the spatial conflict, as little side chain movement is possible for Val. Although the binding site is less crowded in α than in β (Fig. S2), the N-terminal part of Eα is even more rigid than that of Eβ (Fig. S3). As these two factors compensate each other, the effects of stereochemical hindrance in both subunits are equally strong. Since B, C, and the N-terminus of E form a rigid block on the distal side of the heme group in each subunit (Fig. S10), the only conceivable solution to resolve the stereochemical hindrance at the ligand binding site is to move the heme group and its anchor F (Fig. 1b, c). In-plane heme motion upon ligand binding has been experimentally observed and extensively discussed [10], [12]. F in human Hb breaks into a shorter F′ and a longer F, which provides flexibility to allow the crucial motion of the heme group and F (Fig. S7a, b). In contrast, the integral F in an invertebrate Hb and the C-terminus of E (Fig. S7c) form a very rigid block, which helps to achieve a functionally required bending point at the middle of E as a different mechanism of cooperativity in the invertebrate Hb requires [11].

If the heme anchor to F were scissored [13], F would be decoupled from the in-plane heme motion, thus the cascade of structural events depicted in Fig. 1 would be broken, and cooperativity would be weakened or lost. If F does not follow the heme towards the center (Fig. 1c) upon ligand binding, T state would persist as observed previously [13]. However, the distal His and Val would not pose strong stereochemical hindrance to the oxygen binding site if the heme were only attached to an imidazole but not F, which would result in constant high affinity [13]. Persistent T and high affinity seemingly violate the quaternary two state theory of MWC, but strongly support Perutz model that the heme anchor transmits the rearrangement of heme conformation to cause quaternary transition of the protein [12]. These observations from Hb mutants with detached hemes are explained simultaneously here by the sequence of events (Fig. 1).

Eα and Eβ move very little relative to each other except a few degrees of increase in interhelix angle upon ligation (Fig. S8a). In contrast, interhelix distance between Fα and Fβ reduces by about 4 Å upon ligation and continuously does so for almost another 2 Å as the structure transitions to R2 state (Fig. S8b). In the meantime, the interhelix angle between the N-terminal sections of Gs in 3/10 conformation [5] reduces by 10° upon ligand binding, and continuously decreases in R2 state (Fig. S9a). However, the C-terminal portions of Gs are embedded in the dimer framework (Figs. S3 and S4) and move little with respect to each other (Fig. S9b). Large angular motions at the N-termini of Gs and immobile C-termini suggest bending of these helices. Gs are pre-bent in T state and straightened in other states (Figs. 1e, f and S9c), which is a key structural feature to ensure that FGs remain stretched when Fs push towards the center of the molecule (Figs. 1d, g and S8b). The tighter turns at the beginning of Gs help the helices to sustain bending in T state and spring back to straight conformation when Fs allow. That is to say, FG is loaded with tension, more so in T state than in other states. This is evident from the extended conformation of FG unlike other loop conformations. I name such straight, tensioned conformational arrangement *stretch* as a unique secondary structure to distinguish from other loop conformations. The motions of Fs, Gs, and FGs are clearly visible in Movies S1 and S2.

Ligand induced changes in main chain H-bond pattern have been partially analyzed previously. However, these changes seem complex and their functional relevance remains unclear [14]. The companion article [5] presents a global analysis of main chain H-bonds based on singular value decomposition (SVD) of helix identification matrices (HIM) and reveals a complete evolution of helix transformation along the reaction pathway T_High_-T-R-R2. As a result of the helix transformation with four additional H-bonds upon ligand binding in R and R2 states, the end of Fβ suffers little unwinding and does not contribute to backlash since its T state conformation is already unwound. The end of Fα does unwind as Fα pushes towards the center, but the backlash is stopped quickly by three additional H-bonds that significantly firm up this part of the structure [5] so that a large concerted motion of F and G can be achieved. The heme group, F, and FG were previously called “allosteric core” [15]. For the reasons discussed here, I suggest extending the allosteric core further upstream to include F′ and EF, and further downstream to the first part of G. Thus the allosteric core comprises all actions upon ligand binding (Fig. S10) with one exception of the C-terminus of each chain (see below). I shall present that intradimer cooperativity of human Hb is achieved via interplay between the allosteric core full of motions and the distal block that consists of rigid B, C, and the N-terminal part of E (Fig. S10).

### Intradimer cooperativity

Ackers et al. demonstrated that any first oxygen binding in a dimer strongly promotes the second oxygen binding in the same dimer, suggesting intradimer cooperativity. Another study disagrees with the strength of such effect [16]. Nevertheless, the first two oxygens tend to bind to one dimer rather than distribute to both dimers [6], [17]. However, this has long been a controversial issue [8], [18], [19], where the key disagreement lies in the molecular mechanism of cooperativity. The molecular code of Ackers asserts that intradimer cooperativity is not caused by quaternary transition, since this transition requires at least one oxygen binding in each dimer. This theory directly contradicts the well-established MWC two-state theory that cooperativity originates from the transition between two quaternary states regardless whether and how many binding events have occurred. I shall present a new structural interpretation and demonstrate that the previously conflicting aspects are in fact strong support of one another.

Let us consider the scenario of species 11 or 12, in which a first oxygen binding event has newly occurred. How do the motions of the allosteric core and the end of H affect the oxygen binding site in the partner subunit of the same dimer (big ? in Fig. 1)? Since the C-terminus of each subunit barely protrudes in the path of F motion towards the center of the molecule (Figs. S8b and S10a), the interhelix angle between Hs decreases upon ligand binding (Fig. S11). Hα connects the allosteric core of α directly to the distal block of β, and Hβ connects the allosteric core of β to the distal block of α (Fig. S10b). These inter-subunit interactions together with the Gα-Gβ backbone contact (Fig. S6) form the majority of α–β interface. Therefore any motion in one allosteric core is coupled to the distal block of the partner subunit by a lever system of Gs and Hs, where the Gα-Gβ backbone contact acts as a solid pivot point. However, this coupling is associated with a factor of demagnification of roughly 4.5 (Fig. S12). On average, a C-terminus moves about 1 Å upon ligation, thus a complete coupling to the N-terminus of H would be less than 1/4 Å, which is hard to observe considering some loss of motion along the way. It was noticed very early on that the space between F and H is reduced upon ligation [12] and removal of the C-terminal residues (1Y0D and 1Y85) greatly affects the cooperative function [20]. This observation was interpreted as evidences to support the importance of salt bridges of Perutz involving the C-termini [12], [21]. The missing C-terminal hooks would decouple H from F, therefore disrupt the connection between two heme sites in α and β, and result in loss of intradimer cooperativity, and subsequently, interdimer cooperativity.

**Figure 3:**
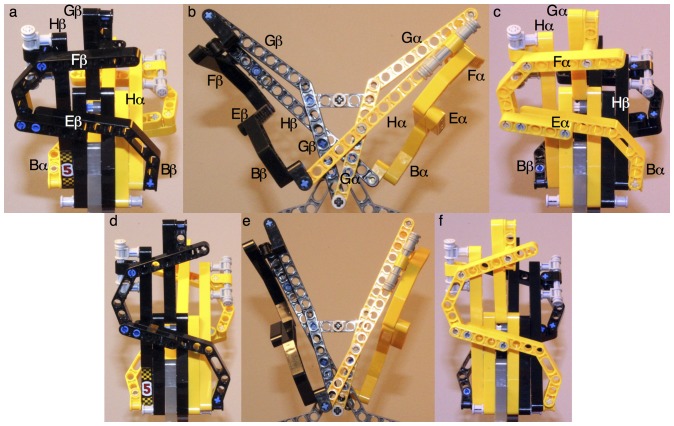
Mechanical model of intradimer cooperativity. Helices represented by solid beams from LEGO^®^ Technic are connected together by pins to allow hinge motions. The main viewing plane (b and e) is parallel to two Gs and two Hs in the same dimer, to which most motions are parallel. The left (a and d) and right (c and f) panels are the views from the left and right, respectively. α and β are constructed identically except that they are in yellow and black. Gray parts do not represent the protein and are required for support. Two Gs are connected at their C-termini to form a V shape (Fig. S10a) as they contact each other with their backbones (Fig. S6). The C-terminal sections of two Gs are locked by a horizontal beam in gray, since these sections are included in the invariant framework (Figs. S3 and S4). Each N-terminal section of G is allowed to bend by insertion of a pivot point (Fig. S9). F and G are connected together at FG. H connects the middle of F to B of the partner subunit. The H to F connection is constructed as a sliding mechanism to reflect the fact that the space between them changes upon ligation. Since B and E are interlocked by a special tight junction (Fig. 2), they are represented by an integral part. However, a hinge to permit the significant F motion joints E and F. Suppose the primary oxygen binding starts from α (species 11). The space between Eα and Fα increases (c to f). The pre-bent Gα is straightened (b to e). The motion of Fα is demagnified at the other end of Hα to move Bβ slightly (Fig. S12). On the other hand, the small motion of Bα is magnified via Hβ to move Fβ towards the center, straighten Gβ (b to e), and opens the space between Eβ and Fβ (a to d). Thus the oxygen binding space in β is cleared and binding affinity increases. In the final R state, two Fs are much closer (Fig. S8b); the angles between two Gs (Fig. S9a) and two Hs (Fig. S11) are smaller (e). For practical reasons, the model is not precisely to the correct scale, and the motions are exaggerated for clarity. Movie S3 shows this model in motion.

Based on these analyses of the dimer structure, a mechanical model is constructed using toy parts from LEGO^®^ Technic to illustrate the lever system of helices (Fig. 3 and Movie S3). In this device, the motions of Fα (Fig. S8b), and subsequently Gα (Fig. S9a), are reciprocated in β. The conformation of a deoxy heme site spontaneously converts to oxy conformation once the other heme site turns to oxy conformation. Two key evidences support this model. First, the SVD analysis of tertiary states [5] shows that the different tertiary states t and r do not mix in a same dimer (see the separate and combined conformational spaces of α and β in the companion article). Second, the less obvious, demagnified motions of Bs and the N-termini of Hs do exist. Fig. S13 shows that although these motions are small in amplitudes, the N-terminus of Hβ clearly moves in coordination with Bα and Eα rather than its own subunit. A more interesting observation is that the Fe-Ni hybrid Hbs in T state [22], [23] (1J3Y, 1J3Z, 1J40, and 1J41) exhibit larger motions in Bα-Eα-Hβ than ligated structures in R state such as 2DN1 and 2DN3. This finding suggests that an allosteric ligand event seems to provoke motions of greater amplitude than an orthosteric event does in its own binding site. This implies that a deoxy subunit is very responsive to a ligand binding in its partner subunit in order to induce a cooperative binding.

In summary, intradimer cooperativity is achieved by a lever system of helices that couples large motions in the allosteric core of one subunit to small motions in the distal block of the other. Two Hs play this vital role on two sides with two C-termini of Gs at the center to act as a pivot point. Both ends of H are indispensable on this long range coupling for the intact function of Hb. However, if intradimer cooperativity does exist, why would a free dimer exhibit no cooperative binding at high affinity?

### Cause of quaternary rotation

Quaternary rotation reaches nearly 15° between T and R states [24], and extends even further in R2 state [25]. Although conformational differences among these states have been studied in great detail, the very question of structure-function relationship of Hb remains unanswered: Why would the relative orientation of two dimers regulate their oxygen binding affinity? Separating the cause and consequence of quaternary rotation is key to establish a sequence of events that leads this dramatic structural rearrangement to interdimer cooperativity. The pinch motion of two FGs towards each other upon ligand binding (Figs. 1d, g and S12, Movies S1, S2 and S3) is the largest structural change within a dimer (Fig. S3). This pinch motion of roughly 3 Å is a simultaneous outcome of the lever system of helices at α–β interface that achieves intradimer cooperativity and subsequently causes the quaternary rotation.

Four couplings between FGs and Cs mediate direct interactions between α1β1 and α2β2. Despite the large pinch motion of FGs, the 3/10 Cs (Fig. S14) do not move closer except a slight motion at the end of Cβ and a small decrease in interhelix angle upon ligation (Figs. S3 and S14). Despite close spatial approximation, a large relative sliding upon ligation occurs between C and FG in a same subunit (Fig. S15). This observation is consistent with the increased interhelix distance and angle between C and F in both α and β upon ligation (Fig. S16).

**Figure 4:**
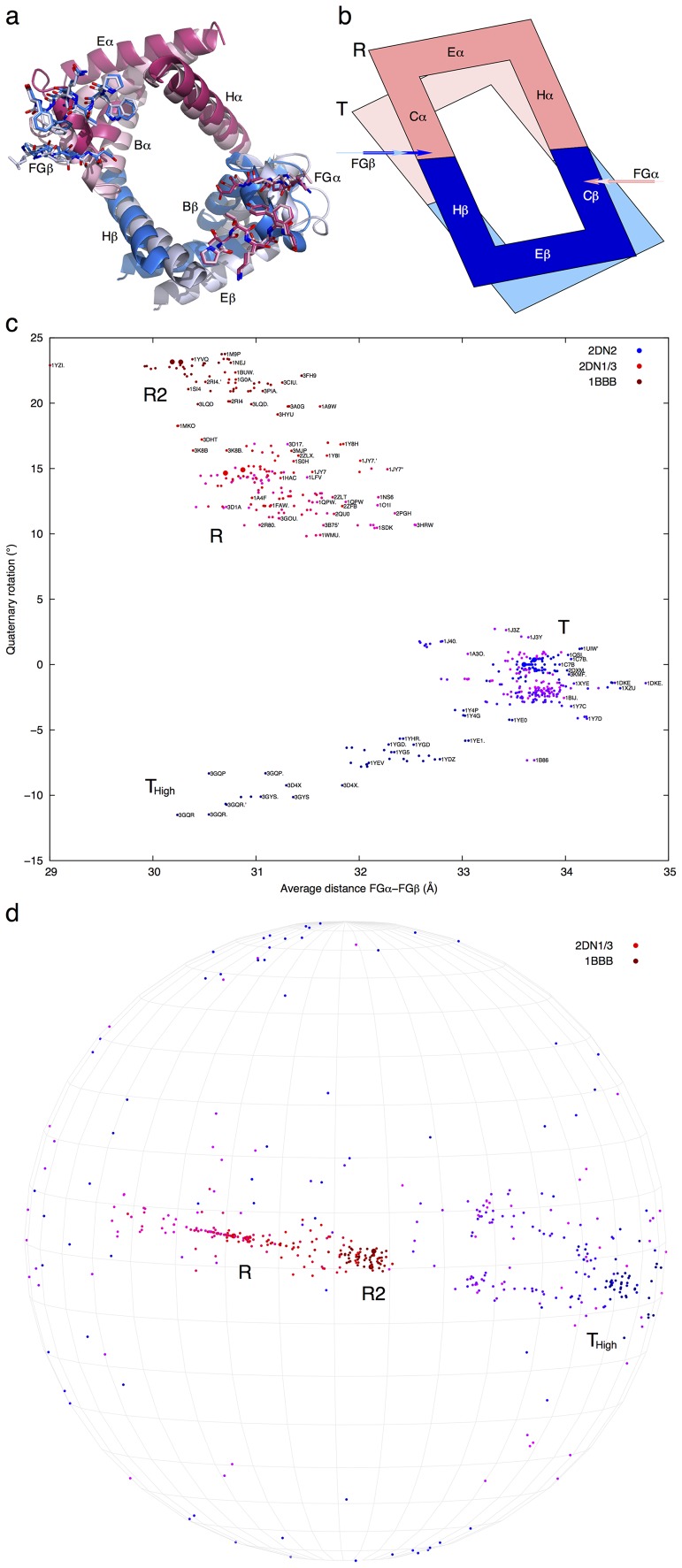
Quaternary rotation. **a**. Experimental structures in T and R states. B to E and H of one dimer are presented by ribbons in background as viewed from the opposite dimer. Cs and FGs of the second dimer are presented by sticks in foreground. α and β are in red and blue colors, respectively. T state is in light colors, and R state after the rotation is in dark colors. **b**. Cartoon of the extracted model. Both the view and the color scheme are the same as in **a**. All helices marked B, C, E, and H of one dimer are represented by a rigid frame of parallelogram to reflect the fact that Cs hold a constant distance between them. FGs of the other dimer are represented by arrows in the directions of the forces applied to Cs. These arrows that pinch towards the center are antiparallel and misaligned to each other. As a result of these acting forces, the lighter parallelogram rotates to the orientation of the darker one. Both FGs move closer to the center. Especially FGβ moves more and slides one pitch of Cα towards BCα. These two panels are reused to illustrate the consequence of the quaternary rotation. Rotation of one dimer as depicted by the ribbons and parallelogram pulls FGs of the other dimer closer to each other. FGα stays fixed with Cβ, while FGβ slips one pitch on Cα towards BCα in order to maintain a constant offset between two FGs. Now the arrows represent the passive motions of FGs resulting from the quaternary rotation. **c**. Correlation of the distance between FGs and the angle of the quaternary rotation. The distance between FGα and FGβ is represented by the average of distances between equivalent main chain atoms in FGs. Each of the tetramers in the structural collection, including those tetramers with α1β1 and α2β2 swapped, is aligned to a reference by least-squares fitting of one dimer to α1β1 of 2DN2. Quaternary rotation angle is determined by least-squares fitting of the second dimer to α2β2 of 2DN2. All least-squares fittings are based on the invariant framework of the dimer (Figs. S3a and S4a). Some of the quaternary rotations in T state are slightly negative because they are opposite of T-to-R rotation when 2DN2 serves as a reference. The quaternary rotations in T_High_ state have large negative values, since these rotations are around very different axes (**d**). Tetramers are labeled whenever possible by small typeface that are only visible on a digital copy. **d**. Orientation of quaternary rotation axis. The axis of each rotation in **c** is from the center of the sphere to a dot on the sphere. Each grid is 10°. The rotation axis sweeps over a range of 60° from R to R2 state in purple to dark red. The negative rotation in T_High_ state in dark blue is 60° away from R2 state in dark red and nearly normal to R state in red and purple. The orientations of small rotations are very unstable seen here as widely scattered blue and purple dots in T state. The coloring of each symbol in **c** and **d** indicates the position of a tetramer in the reaction trajectory T_High_-T-R-R2 (see Figure 2 of the companion article).

Pinch of FGs in one dimer and fixed Cs in the other are not compatible with each other when they are directly coupled together. The incompatibility generates forces. Resolution of such incompatibility requires a rotation of the pair of Cs (Figs. 1k, l and 4a, b). Here I reveal that the rotation is first driven by two misaligned antiparallel forces exerted on Cs of the deoxy dimer by FGs of the opposite, ligated dimer. Second, the direction of each force is not normal to the sides of the rigid parallelogram structural frame where two Cs are located. Instead, the forces are applied at two positions that are on a diagonal of the parallelogram frame. A rotation of the parallelogram will allow two FGs to move closer, one of them to stay locked with a C (Fig. 1k), and the other to slide on the other C (Figs. 1l and 4a, b). The structural basis of these critical interactions between C and FG are the well-known flexible hinges at Cβi-FGαi and ratchet switches at Cαi-FGβi [10], [12] (Fig. S1).

Direct calculation of the distances between FGs and the quaternary rotation angles for all tetramers in the structural collection shows that these two parameters are strongly (anti)correlated (Fig. 4c), which further indicates that the pinch motion of FGs is the direct cause of quaternary rotations (Movie S4). This correlation was previously observed [14], but the pinch of FGs caused by straightening of Gs was described as rigid body motions of α and β, and termed intradimer bending [26], [14]. Although the distance between FGs is a continuously distributed parameter, the angle of a quaternary rotation exhibits a 7° gap that clearly distinguishes R state from T. This discontinuity in quaternary rotation again demonstrates the allosteric taboo gap found in SVD analysis of distance matrices [5]. The orientation of the quaternary rotation axis is also strongly influenced by the distance between FGs (Fig. 4d), and is very different for T_High_ compared to R and R2 states [14] (Fig. 4d). The companion article presents a molecular mechanism of T_High_ state [5], which explains the different orientation of its quaternary rotation and elucidates that its high affinity is due to the same factor that is responsible for high affinity in R and R2, as the pinch motion of FGs has also occurred in T_High_ (Fig. 4c).

The motions of two FGs resulting from ligations in α and β (Figs. 3 and S15) effectively integrate two chemical signals of ligand binding. The total motion drives a quaternary rotation. Hence a partially ligated state would feature incomplete pinch. The deoxy dimer opposite of a singly ligated dimer does not seem to sense whether α or β is ligated. Would a partial pinch cause a quaternary rotation to stop halfway? The Fe-Ni hybrid Hb structures cross-linked at Lys82 between two βs [22], [23] are available to address this question. These structures (1J3Y, 1J3Z, 1J40, and 1J41) exhibit T state dimers closest to R state in the correlation plot of rotation angle vs. distance between FGs (Fig. 4c). SVD analysis of distance matrices of dimers [5] also shows that these hybrid structures have moved out of the tight cluster of typical T state but linger around in the vicinity, which suggests that the sum of a partial pinch due to a single ligation is insufficient to cause complete quaternary rotation, but does drive the rotation towards R state. Due to the 7° taboo gap, a quaternary rotation never stops at a midpoint (Fig. 4c). The fundamental reason for the discreteness of quaternary states in MWC is explained below. It would cost even more free energy to undergo a quaternary rotation from a singly ligated tetramer 11 and 12 to form two singly ligated dimers in species 22, 23, or 24 than what a primary oxygen binding costs, that is, negative cooperativity [17], which may explain why cross-linking between two βs [22], [23] is required to achieve such unusual structure.

### Consequence of quaternary rotation

It is clear that the quaternary rotation leaping over the allosteric taboo gap [5] marks the transition between T and R states (Fig. 4). However, identifying the large pinch motion of FGs being the direct cause of the quaternary rotation still does not answer the very fundamental question – why would oxygen binding affinity be modulated by relative orientation of two dimers? It is important to point out that any rotation of α1β1 relative to α2β2 is exactly equivalent to that of α2β2 relative to α1β1 in terms of both angle and direction of the rotation. From a remote perspective, if α1β1 rotates clockwise, α2β2 seems to rotate counterclockwise. But their rotations relative to each other occur simultaneously with the exact same angle and direction. Therefore the figures that illustrate the cause of the quaternary rotation can be reused to explain the consequence (Fig. 4a, b), except with different perspectives. As FGs get closer in a fully ligated dimer in species 21, the deoxy dimer starts to rotate (Figs. 1k – n and 4a, b). It is completely equivalent to consider the ligated dimer rotating the same amount relative to the deoxy dimer, which pulls FGs of the deoxy dimer closer to each other by the rigid Cs of the rotating ligated dimer (Figs. 1o, p and 4a, b). Due to the stretch conformation of FG and the pre-bent Gs, little motion loss is possible along these chains from the heme anchor to the beginning of G. Consequently, two Fs of the deoxy dimer are pulled closer to each other as well. Each F carries the heme group that anchors on it towards the center of the molecule, a motion that effectively vacates the oxygen binding sites from the occupation of distal His and Val, and increases their oxygen binding affinity. The effectiveness of this process is facilitated by the immobile Es that are tightly held by Bs (Fig. 2) so that they cannot follow the motion of Fs. Once the third ligation occurs (species 31 or 32), the mechanism of intradimer cooperativity once again takes over (Figs. 3 and S10). The oxygen binding affinity at the last site returns almost to the level of a free globin [27].

The cause and consequence of a quaternary rotation seem identical – the distance between a pair of FGs decreases by 3 Å. However, the key difference is that the cause is an active pinch motion of FGs in one dimer while the consequence is a passive closing of FGs in the other. Although the quaternary rotation is the most significant structural event along the reaction pathway of Hb, it is not the end result; rather it mediates two seemingly identical events in two dimers at the mid point of the pathway. The quaternary rotation is the molecular mechanism to maintain symmetry between two dimers once the symmetry is broken by partial ligand binding or releasing.

**Figure 5:**
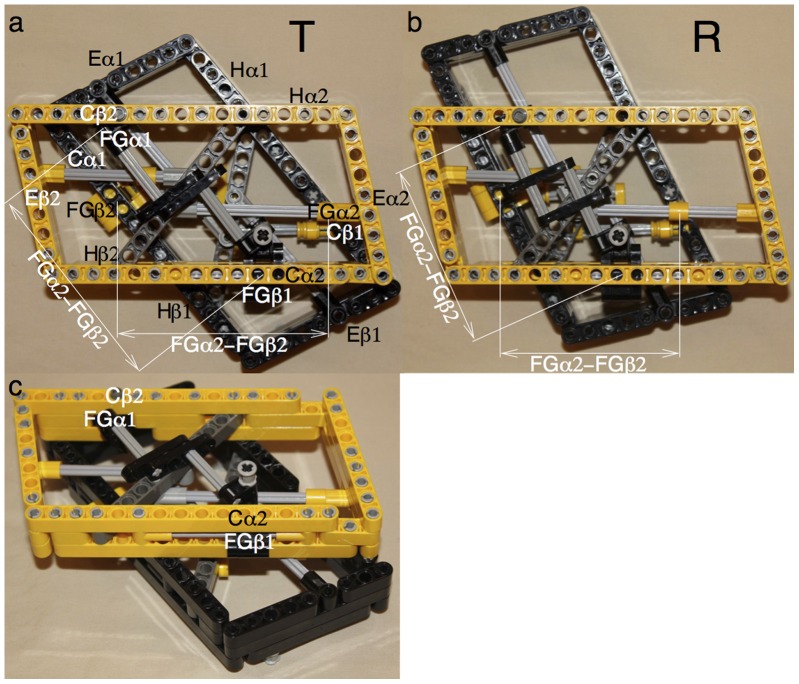
Mechanical model of quaternary rotation. The solid parallelogram of each dimer formed by B, C, E, and H (Fig. 4a) is constructed using beams from LEGO^®^ Technic. The two dimers are identical but in black and yellow, respectively. Gray rods act as tracks that guide the moving parts. Two FGs in each dimer can slide along the two parallel but misaligned gray rods. FGβ1 in black and FGβ2 in yellow can slide along the side of Cα2 and Cα1, respectively. Movie S5 shows this model in motion. **a**. In T state, FGs in both dimers have greater distance between them. **b**. In R state, FGs in both dimers move closer and one dimer rotates relative to the other. **c**. The same model shown at an angle to reveal more detail.

Another LEGO^®^ model is constructed to illustrate this reciprocating pinch-rotation-pinch mechanics (Fig. 5 and Movie S5). Two identical models of dimer are interconnected so that pinch of two parts of either model causes the other model to rotate and subsequently brings the equivalent two parts closer in the rotating model. This device demonstrates that quaternary rotation is not the final outcome, but a mechanical strategy to couple two identical linear motions in two dimers. Force, and more importantly, work is transmitted from one linear motion to the other.

## Discussion

In homodimeric Hbs from invertebrates, cooperative motions are transmitted through a pair of specialty pliers [11]. Dyadic symmetry in either deoxy or ligated states is quickly regained once a new ligand event knocks the dimer out of symmetry temporarily. Within the context of invertebrate Hbs, the pliers model seamlessly unified the sequential model by Pauling-KNF [3] and the symmetry model by MWC [4]. Would all major theories unite on cooperative mechanism of tetrameric Hb, the paradigm of protein allosteric regulation?

In essence, an oligomeric allosteric system is intrinsically capable of interconverting among a small number of discrete states while preserving inter-subunit symmetry [28], [29]. Over the past decades, this MWC allosteric theory has been proven very successful except that atomic level interpretations are missing for two key assertions, namely symmetry conservation and discreteness of states. Other allosteric theories attempt to provide mechanistic details with the most noticeable ones being the microstates [30], [6], [17] and tertiary two-state [7], [8] theories. Ackers et al. summarized carefully resolved possible microstates in a symmetry rule of quaternary transition. The microstates theory is essentially a modernized version of the sequential model by Pauling-KNF [3], but again lacks a structural interpretation of the mechanism of the molecular code. The tertiary two-state theory can be considered as a more relaxed MWC that tolerates two tertiary structures that disobey symmetry during quaternary transition. It updates MWC and attributes oxygen binding affinity to the tertiary structure of each subunit instead of the quaternary state of the assembly. Nevertheless, the common belief is that it is inevitable to study intermediates, which play no role in MWC, for better understanding of reaction mechanism. In this section, I shall discuss the findings of this and the companion article [5] in the greater context of protein allostery that has been evolving for past decades [8].

### Symmetry conservation

This simple beauty of MWC asserts that inter-subunit symmetry is preserved in all states. Cooperative function originates from symmetry conservation, since it requires all subunits to undergo the same changes as soon as a process is triggered by a primary event in one of the subunits. This work on human Hb, together with the previous example from an invertebrate dimeric Hb [11], provides the long sought atomic level structural mechanisms of symmetry conservation that strongly support MWC. It becomes clear that markedly diverse mechanisms can be employed to achieve symmetry conservation. For example, in the dimeric Hb from invertebrates [11], the opening and closing of E and F in one subunit are directly coupled to the partner subunit to produce the same motion. Within a human Hb heterodimer, motions in the allosteric core of one subunit are coupled to the rigid distal block of the partner subunit via a lever system of helices (Fig. S10). The large pinch motion of FGs resulting from intradimer cooperativity in human Hb subsequently drives the quaternary rotation required for interdimer cooperativity (Figs. 4 and 5). The human Hb tetramer, considered as a homodimer of two heterodimers, employs a circular motion of a large quaternary rotation to couple two identical linear motions between two heterodimers. In all cases, a cooperative event reinforces structural changes in all subunits including the one in which the primary event occurs. A common principle of symmetry conservation lies in the synergetic motions among partner subunits intrinsically warranted by the mechanics of each protein.

### Discreteness of states

**Figure 6:**
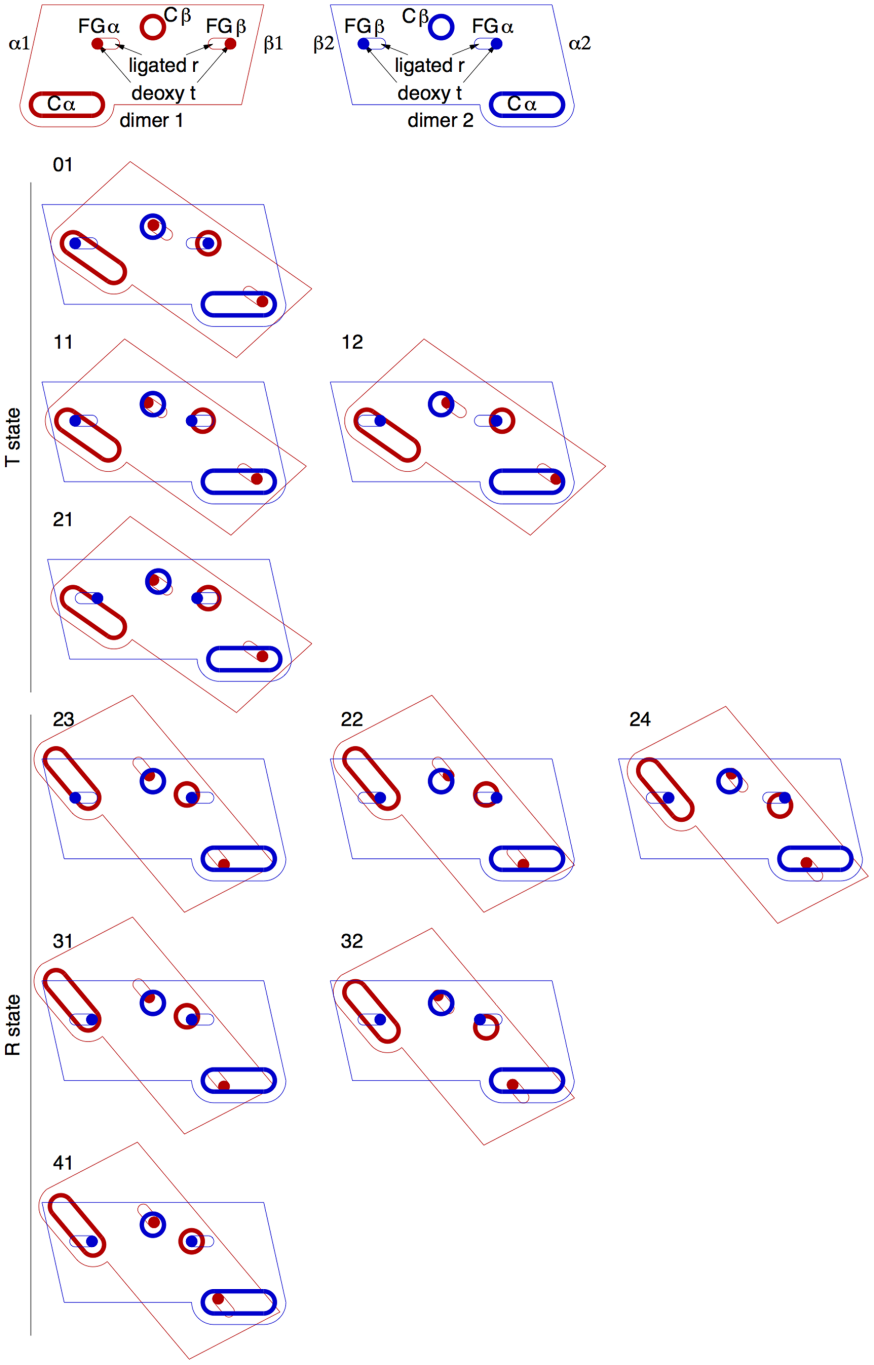
Schematic drawing of microstate structures. Each dimer is represented by a parallelogram (Fig. 4a). FGs, as indicated by the solid dots sliding in the slots, are positioned at the far ends in deoxy state and the near ends in ligated state. The circular and elongated thick loops stand for Cβ and Cα, respectively. When two dimers are engaged, the circular loops matching the dots represent hinges at Cβi-FGαi. The elongated loops allow FGβi to slide between two ratchet positions on Cαi.

MWC also asserts that an allosteric system features a small number of quaternary states, most often two, that are discrete in both structure and function. Although the discrete T and R states of Hb differ very little in free energy [31], they do markedly in structure. This discreteness is unambiguously demonstrated by the approach of meta-analysis here as the wide allosteric taboo gap [5] (Fig. 4c). Is the discreteness in states functionally relevant? If so, how does the structure of Hb achieve discrete quaternary states? The prominent allosteric taboo gap collectively demonstrated by hundreds of Hb structures warrants that the four oxygen binding sites favor either all vacant or all occupied. Such discrete states of all-or-none are analogous to coal-transporting dump trucks. The most efficient way to transport is to load fully at the source and to dump completely at the destination. In case of Hb, partial loading and unloading is prevented by the intricate structure of this oxygen carrier. Although the companion article [5] discusses several incidental details that support discreteness of states, the most fundamental reason is rooted in the mechanism of quaternary transition presented above. Each oxygen binding requires a displacement of F and FG, as depicted by a sliding pin in a slot in the schematic drawing of the microstates (Fig. 6). By mechanically coupling the motions of four pins together, it is required to displace all four pins at the same time of a quaternary rotation in order to maintain perfect fitting between Cs and FGs. Any combination of deoxy and ligated subunits in a tetramer leads to poor fitting between Cs and FGs regardless of any quaternary rotation. Thus all mixtures of t and r tertiary structures in the intermediate microstates must be short-lived. Each tetrameric molecule must quickly settle in either species 41 or 01, that is, all or none. The efficiency of oxygen transport by Hb is achieved by the only two energetically equivalent occurrences of geometric fit among the four small interdimer couplings with very limited freedom as specifically defined by the pinch motion of FGs and two ratchet positions on Cα but not Cβ. This is the structural basis why oxygen binding affinity does not depend on whether and how many ligand binding events have occurred, but on the two discrete states.

The companion article [5] also discusses why α and β chains differentiate in tetrameric Hbs and provides a molecular mechanism for the abnormal homotetrameric Hbs, which is branched out from the mechanism of the discrete quaternary transition presented above. In time-resolved experiments, the short-lived intermediate species are produced synchronously thus can be captured as they reach significant concentrations. These asymmetric species that would fill the allosteric taboo gap do not belong to either of the discrete states and are hypothesized in the companion article [5]. The further extended tertiary two-state theory [7], [8] would be required for proper modeling of these intermediate species that usually do not accumulate in blood nor in static crystals.

### Molecular code

This understanding of discreteness in quaternary transition also elucidates the mechanism of molecular code. The molecular code apparently discards the two-state equilibrium theory of MWC and unambiguously categorizes the doubly ligated species 21 in T state if the first two ligations occur cooperatively in a same dimer. But the other doubly ligated species 22, 23, and 24 were found in R state if the first two ligands are separated in both dimers. This is completely comprehensible given the mechanical model of the quaternary rotation presented here. The doubly ligated dimer in species 21 would generate the pinch motion of FGs. However, the deoxy dimer maintains a large distance between FGs. If the quaternary rotation occurs in 21, two FGs in the deoxy dimer would close; if it does not, two FGs in the ligated dimer cannot pinch. The quaternary rotation is supposed to couple the linear motions between two pairs of FGs (Fig. 5 and Movie S5) and cannot occur in such asymmetric, jammed device until the FGs in the deoxy dimer are allowed to close more by binding of a third ligand to form species 31 or 32. If both dimers are singly ligated (species 22, 23, or 24), both pairs of FGs close partially with equal amount (see discussion above on the Fe-Ni hybrid Hbs). The quaternary rotation would proceed smoothly without conflict in these symmetric species. However, these species violate the intradimer coupling mechanism (Fig. 3), since such singly ligated dimer presents a jammed lever system at the dimeric core, thus costs extra energy to form [17]. The key disagreement here is whether the second binding event to form two singly ligated dimers is barely cooperative [16] or negatively cooperative [17]. Despite any potential experimental errors, the molecular code of Ackers does not discard MWC but reflects the molecular mechanism of symmetry conservation of MWC. The microstates theory is indeed a detailed implementation of MWC quaternary two-state theory. These theories do not constitute any conflict in the mechanistic understanding presented here. However, this is only possible when the available Hb structures are interpreted jointly to provide a correct structural foundation [5].

### Allosteric signal transmission

Molecular mechanism of long-range signal transmission through protein as required by allosteric regulation is usually described as conformational changes in a cascade of key side chains along a path or through a network. This path in Hb is supposed to connect one heme site to the other, or at least to the interface of subunits so that changes of Fe position in one heme can be indirectly “felt” by a neighboring subunit through a chain of dominos [32], [10], [33], [21]. The cooperative machinery I present here and the pliers model of the dimeric Hb [11] depart noticeably from the traditional dominos concept of protein allostery. Here protein function of physical, mechanical, or geometrical nature that is distinct from enzymatic reactions of chemical nature is dissected into mechanical parts and reverse engineered as a concerted machine. In these mechanical models, some helices perform as rigid levers, some others feature hinges in the middle that allow kink or bending. Energy stored in bent helices may be released to facilitate structural conversion. An intact part of a helix may be temporarily and partially deformed to provide a mechanical buffer [5]. Two helices may be jointed together via a very tight dovetail structure that often involves direct backbone contacts. Such tight joints are responsible for motion transmission without backlash, since they only permit slight pivoting, but do not tolerate relative sliding. Parts of a helix may partially overwind or unwind to interconvert between 3/10, α, π, and even +6 forms [5], which change mechanical property of the helix. In addition, extended stretch conformation sustains tension like a string. These mechanical parts work together with the well-known flexible joints and ratchet switches in a machine to achieve protein allostery. In such protein machinery, the protein main chains play a role of infrastructure that facilitates the leading function of chemical nature, for example here oxygen binding. This view of protein allostery is in sharp contrast to the dominos concept that mostly involve side chain movement along a path or in a network.

Both motion and immobility of mechanical parts are equally important to allosteric functions. More than often, structural analyses focus on motions but largely ignore the role of immobile parts in a protein structure. I here argue that without rigidity in a structural framework, moving parts cannot extend their motions to full ranges. For example, the α–β interface must be very stable to ensure the large pinch motion of FGs. The distance between Cs must stay constant to generate sufficient forces for the quaternary rotation to occur. Tight dovetailed junctions between helices serve as an effective strategy to prevent adverse backlash in protein structures. However, motion loss remains highly probable in a folded polypeptide chain. For example, the addition of several H-bonds upon ligand binding near heme anchor sites controls possible motion loss [5]. The distance between two Fs in a dimer decreases by 4 Å in R state compared to that in T (Fig. S8b). However, the distance between two C-termini in the same dimer decreases only 2 Å (Fig. S12). Motion is lost in the buffer space between F and C-terminus [12]. Removal of C-terminal residues made this motion loss unbearable [20].

What is the carrier of signal that is transmitted over long range through protein? Motion is the usual answer as it is more tangible from crystallographic observations and many other experimental methods. However, the mechanical models presented here imply that force is the real carrier of a structural signal while motion is rather an indicator of force. That is to say, it is work that is transmitted from one part to another in a structure. Therefore, force and motion are respectively the cause and consequence of structural changes. The chief task to achieve a mechanistic understanding of protein function is to identify undetected forces from observed motions. Transmission of a greater force is often accompanied by a smaller motion in a lever system. Subtle motions may not be readily visible given certain noise level. For example, when the large pinch motion of two FGs in one dimer exerts forces on two Cs in the opposite dimer, long-range transmission of force over more than 30 Å through the rigid dimeric core requires no obvious motion. Thus tracking down motions along a path or through a network of allosteric interaction under the traditional concept is often difficult.

I recognize that energy calculations at thermodynamics and single molecule levels are required to support the reverse engineered model. These calculations fall beyond the scope of these two companion articles. The mechanical model proposed here would provide a structural basis for these detailed calculations.

### Oxygen binding affinity

Finally, back to the most fundamental question in Hb function, what is the structural origin of oxygen binding affinity? Given a constant reactivity of a free diatomic oxygen with a five-coordinated high-spin ferrous Fe(II) out of the heme plane to form a six-coordinated low-spin Fe(II) in the heme plane that covalently links to the oxygen, the binding affinity of this reaction in a protein environment is conversely affected by two competing factors. First, the accessibility to a deoxy Fe enhances oxygen binding affinity. Second, the ability of a bound oxygen to escape from the oxy Fe diminishes affinity. MWC attributes the difference in affinity to the distinct quaternary states. This work provides an atomic level molecular mechanism that elucidates the relative orientation of two dimers is mechanically related to the conformation of the allosteric cores where oxygen binding sites are located. This mechanism also links the quaternary states to the tertiary structures. Each tertiary state dictates the affinity in its own binding site as the tertiary two-state theory correctly asserts. The open FGs of two tertiary t structures position the Fs and heme groups up against the distal His and Val in Es, so that the deoxy Fe are in low affinity because they are not readily accessible by oxygen.

The closer FGs of two tertiary r structures, on the other hand, shift the Fs and heme groups inward. As the oxygen binding sites are cleared, their affinity increase. Hb Bristol-Alesha [34] and Toms River [35] are Val67Met mutants in β chain and γ chain of fetal HbF, respectively, where the Met may be post-translationally converted to Asp. Hb Bristol-Alesha is unstable that causes hemolysis, and Hb Toms River has low affinity that causes neonatal cyanosis and anemia. The larger side chains of Met and Asp reduce the accessibility of deoxy Fe and lead to low affinity [35]. The mechanical model presented here predicts that oxygen binding to these Hb mutants would require extra large pinch motions of FGs to clear the binding sites, which may result in completely straight Gs in R state (Fig. S9c). The tetrameric assembly would become less stable and two dimers would have greater chance to dissociate without bending of Gs [5].

It is interesting to note that Hb from deer mice from terrains of high altitude features Ala57αGly and Ala62βGly variations with increased affinity [36], [37]. Why would a methyl group here affect oxygen binding affinity? These variations at E6 right next to distal His at E7 are expected to reduce the stiffness of this region thus renders less stereochemical hindrance on the oxygen binding site imposed by the distal His, resulting in higher affinity. More generally, a number of positions on E around distal His could vary between Gly and non-Gly [37]. It appears that these variations are responsible for fine-tuning the stereochemical hindrance that the distal His imposes on the oxygen binding site. However, E8 Gly never changes in either chain, because it is required to form the rigid B–E dovetail crossing (Fig. 2) essential for generating the pinch motion of FGs.

The global analysis of Hb structures in PDB and the reverse engineered model also provide the long-sought structural basis for the mechanisms of allosteric effectors [38]. Several allosteric effectors bind inside or at the entrance of the central cavity. For example, the indigenous allosteric effector 2,3-bisphosphoglycerate (2,3-BPG or DPG) is wedged in between two β chains in T state to promote low affinity (1B86) [39], [40], because the high affinity R state would reduce the gap between β chains. Another larger compound inositol hexakisphosphate (IHP) has not only been wedged in the gap between β chains but also shimmed the gap slightly wider than usual (3HXN). Both 1B86 and 3HXN are located in the T cluster of the computed trajectory but at spots further away from R than that of a more typical T structure (see Figure 2a, c of the companion article). The IHP complex 3HXN behaves more unusual than the DPG complex 1B86. This illustrates the structural basis of these allosteric effectors as they alter oxygen binding affinity in T state. The other drugs (2-[4-[[(3,5-dimethylanilino)carbonyl]methyl]phenoxy]-2-methylpropionic acid or RSR-13 [41], 1,3-phenylene[bis(2-{4-[(aminocarbonyl)-methyl]phenoxy}-2-methylpropionic acid)] or TB5-27 [42], and 2-[4-(3,5-dichlorophenylureido)phenoxy]-2-methylpropionic acid or L35 [43]) lower the oxygen binding affinity by filling the central cavity. When two molecules of L35 bind inside the cavity, the ligand induced quaternary change still proceeds (2D60 and 2D5X). A third L35 would prevent Hb from shrinking the central cavity, thus would keep Hb in T state even with some ligand bindings (2D5Z). However, a unique allosteric effector bezafibrate (BZF) that also lowers the affinity binds on the surface of α globin, and attaches on both Eα and the side of the heme (1IWH). As a consequence, the α heme group is held closer to distal Eα even in R state [44]. Thus stereochemical hindrance at the oxygen binding site in α increases, and a bound oxygen has a greater chance to be squeezed out.

Hb locked in T state either by crystal lattice [45]–[47] or in gel [48] loses both intra and interdimer cooperativity. Intradimer cooperativity based on the lever mechanism requires the quaternary rotation as much as interdimer cooperativity does. Therefore, a non-cooperative free dimer [5] and the loss of cooperativity in crystal and gel cannot invalidate intradimer cooperativity.

## Materials and Methods

These two companion articles deduce an atomic level molecular mechanism of the quaternary rotation between two dimers in a Hb molecule, thus provide a structural basis of MWC allosteric theory by elucidating its key principles, symmetry conservation and discreteness of states (circle in Fig. S17). The main conclusion of these articles reveals the origin of oxygen binding affinity in Hb, while the methodological advance suggests much broader application of the analytical strategy for joint evaluation of increasingly greater number of known structures in PDB. The diverse topics and the logic of reasoning presented in these two articles are illustrated by the directed graph in Fig. S17, which may serve as a roadmap to help navigate through the multitude of evidences that support each of the intermediate findings.

At the top of the graph (Fig. S17), three main analytical strategies lead the way, distance matrix, SVD, and parameterization of helix. Due to the choice of these methods, structural alignment that often plays a critical role in other analyses is largely avoided here. Among the structural findings, the lever system of helices at the dimeric core of Hb, the pinch motion of two FGs, the t tertiary state, and several r tertiary sub-states occupy the central position in the graph (oval in Fig. S17) suggesting their importance in connecting multiple structural evidences to the final conclusions in the circle. These central findings directly support intradimer cooperativity in molecular code.

A cluster of nodes on the left side of the graph consists of the subjects that involve the detailed mechanism of the transition across the allosteric taboo gap. These findings also support the final conclusions in the circle from another perspective.

### Invariant structural framework

The computational problem of optimal alignment or superposition of multiple structures was considered previously [49], [50]. However, this is only a sub-problem given the purpose of the alignment – to identify unbiased structural motions or differences embedded in a structural ensemble. I here pose a different problem with greater priority – how to divide a structure into two sections, neither is necessarily consecutive, with the first as large as possible and the other as small as possible so that the first section can be optimally aligned as described previously while the second section is least aligned. In other words, what is the relatively invariant and broad-based structural framework, and what is the most localized but maximized signal in structural variation? In case of structural analysis of Hb, this problem was posted long ago and solved multiple times empirically [10], [11], [51]. The previous practices do not suggest a proper computational solution, but they support the argument that structural bisection is required during structural alignment. A proper computational solution to this combinatory minimization problem lies outside the scope of this paper. However, I suggest and implement a practical approach based on distance matrix [52], [53] that extends from the subjective structural bisection previously used [10], [11], [51]. After an invariant framework and a mobile section of a structure are identified, they can be further bisected in the same fashion if necessary.

An rmsd matrix contains rmsd values calculated from the corresponding items of a set of distance matrices of the same size. The column (or row) average of rmsd matrix measures the mobility of the structure at a specific atom. Fig. S3 shows the rmsd matrices calculated from the collection of αβ, α, and β. The rmsd matrix of αβ (Fig. S3a) shows that the dominant motion within a dimer is between FGs of α and β relative to each other. By comparing rmsd matrices of α and β (Fig. S3b, c), it is clear that they behave differently and β moves more extensively than α in general.

Rmsd matrix is an easy way to analyze a structural collection jointly, and it provides an overall view of structural mobility. On the other hand, it is an equally important usage of rmsd matrix to identify the rigid framework of protein structures that does not undergo large conformational change regardless of the states of a protein. The identified rigid framework will then serve as the alignment segment of least-squares fitting in structural comparison [11]. The structural segments as part of a rigid framework must first be internally rigid, that is, all rmsd values are small in the squares on the major diagonal. Second, these segments must display little or no motion relative to one another, which is indicated by small rmsd values in all rectangles off the major diagonal. The rigid framework of αβ is identified as a large part of Bα and BC loop (residues 26–41), a small but important part of Eα (residues 57–62) that contains distal His58α and Val62α, the most of Gα (residues 100–113), a part of GH and the first part of Hα (residues 117–132), a part of Bβ (residues 26–35), and a part of Gβ to Hβ (residues 106–137) except the residue 120 in GH. This framework has an overall rmsd value in 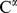 motion of 0.22 Å across more than 500 dimers in the collection. The framework of αβ is largely based on the very stable interface between subunits [10] with slight extension (Fig. S4a). This identification of invariant framework from a large number of structures differs modestly from the previous identifications based on a few structures [10], [51].

If α and β are considered separately, their frameworks extend a little further (Fig. S4b, c) with the overall rmsd values of 0.21 Å (Fig. S3b, c). However, no rigid framework can be found for the tetramer due to the large quaternary change.

### Parameterization of helix

Like inter-atomic distances [5], relative geometric relationship between secondary structural elements such as interhelix distance and angle are also independent of any coordinate system, thus structural alignment can be avoided when analyzing these relative relationship. Here I develop an analytical formula to express main chain conformation of a helix. Each straight helix can be considered as a combination of four helices of N, 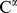, C, and O that share a same axis, a same pitch *s*, and a same angular turn per residue, but each has a slightly different radius. The Cartesian coordinates of an atom of type A in a straight helix with an orientation cosine ***n*** of its axis can be written as
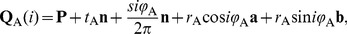
(1)where ***a*** and ***b*** are other two orientation cosines normal to each other and both orthogonal to ***n***. They can be obtained by ***a*** = ***n***×***c*** and ***b*** = ***n***×***a***, where ***c*** is an arbitrary orientation cosine that is not parallel to ***n***. The axis of the helix passes a point ***P***. *r*_A_ is the radius of the helix for atom type A. The average of these four radii represents the radius of the overall helix. *t*_A_ and *φ*_A_ are small translation along the axis and phase shift of the A-helix, respectively. Usually, let *t*_C_^α^  = 0 and *φ*_C_^α^  = 0. *i* is the residue ID to which atom A belongs. A total of 18 parameters are sufficient to describe all main chain atoms in a straight helix. A bent helix can be treated as two or more straight helices.

Least-squares fitting of all 18 parameters will provide the location, orientation, radius, pitch, and angular turn per residue accurately. Interhelix distance and angle can be calculated subsequently as the distance and angle between two axes.

## Supporting Information

Figure S1 **Four sides of Hb in T and R states.** Four E–F pairs form four sides of Hb tetramer with the parallel, opposite sides from a same dimer. **a**. The four sides shape like a diamond in T state. **b**. They transform into a square in R state.
(TIFF)

Figure S2 **Deoxy binding site conformation.** Oxygen-bound heme group in white is placed in deoxy binding site to show conflict of oxygen with distal His and Val. **a** and **b**. α in pink. **c** and **d**. β in light blue. **a** and **c**. Viewed from the interior of the molecule. **b** and **d**. Viewed from the exterior of the molecule.
(TIFF)

Figure S3 **Rmsd matrices.** Larger rmsd values in darker blues indicate greater structural mobility. Small values in pale green indicate invariant structural segments. Black squares on the major diagonal outline the internally rigid structural segments automatically identified. Black rectangles off the major diagonal mark the inter-segment variation. All segments must exhibit both small internal variation and small inter-segment variation to be part of the invariant structural framework. That is to say, the submatrix outlined by the black squares and rectangles must have a small average value. An automated procedure developed here evaluates the penalty upon expanding the submatrix and the saving gained by shrinking the submatrix. **a**. αβ. **b**. α. **c**. β.
(TIFF)

Figure S4 **Invariant structural framework.** The invariant framework identified from 560 structures is in green. The other parts of α are in pink and those of β in light blue. **a**. αβ. **b**. β. **c**. α.
(TIFF)

Figure S5 **Parameterization of B and E.** Helices from α and β are in red and blue, respectively. Triangle and diamond represent B and E, respectively. All helical parameters are plotted in the sequence of the reaction trajectory along T_High_-T-R-R2 as identified in the companion article [5], and this applies to all similar figures below.
(TIFF)

Figure S6
**Backbone contact between Gα and Gβ.** Ala111α 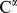 and O are less than 3.5 Å away from Ala115βO and Gly119β 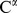, respectively. Ala110αO-His116β 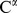 is slightly greater than 3.5 Å.(TIFF)

Figure S7 **Comparison of E–F.** **a**. Human α. **b**. Human β. **c**. Invertebrate dimeric Hb.
(TIFF)

Figure S8 **Parameterization of E (a) and F (b).** Helices from α and β are in red and blue, respectively. Interhelix parameters between α and β are in black. See also Fig. S5 legend.
(TIFF)

Figure S9 **Parameterization of G.** Helices from α and β are in red and blue, respectively. Interhelix parameters between α and β are in black. **a**. N-terminal section of G in 3/10 conformation. **b**. C-terminal section of G in α conformation. **c**. Bending angle between N- and C-terminal sections. Notice that the 3/10 helices have significantly smaller diameter, but larger pitch and turn per residue compared to α helices. See also Fig. S5 legend.
(TIFF)

Figure S10 **Allosteric core and distal block.** α and β are in warm and cool colors, respectively. The allosteric cores and distal blocks are in darker and lighter colors. **a**. Side view with dimer interface facing up. **b**. Top view directly into the dimer interface from the opposite dimer.
(TIFF)

Figure S11 **Parameterization of H.** α and β are in red and blue, respectively. Interhelix parameters are in black. See also Fig. S5 legend.
(TIFF)

Figure S12 **Demagnification of motion transmission.** α and β are in warm and cool colors, respectively. Deoxy and ligated states are in light and dark colors.
(TIFF)

Figure S13 **Relative motion between Bα-Eα-Hβ and Bβ-Eβ-Hα.** Each panel is labeled by the source of a dimer structure. Helices are labeled on the first panel only. All structures are aligned to 2DN2-α1β1 by least-square fitting of Bβ-Eβ-Hα on the right side of each panel. Atomic displacements of Bα-Eα-Hβ on the left side of each panel are marked by green arrows. Each arrow is three times as long as the real displacement.
(TIFF)

Figure S14 **Parameterization of C.** α and β are in red and blue, respectively. Interhelix parameters are in black. See also Fig. S5 legend.
(TIFF)

Figure S15 **Small interdimer couplings Cs and FGs.** C and FG in a same subunit run antiparallel to each other and both are roughly parallel to the heme plane. Deoxy and ligated structures are in light and dark colors, respectively. **a**. α. **b**. β.
(TIFF)

Figure S16 **Parameterization of C and F.** Helices from α and β are in red and blue, respectively. See also Fig. S5 legend.
(TIFF)

Figure S17 **A directed graph of the topics in the companion articles.** The main topics presented in these companion articles [5] are linked by the directed edges that indicate the direction of reasoning. The overall conclusion from these two articles is the molecular mechanism of a cluster of nodes in the circle. The oval at the center of the graph outlines the key findings that play a central role in the analysis.
(TIFF)

Movie S1 **Pinch motion from a side view.** The pinch motion of αβ is viewed from a sideway so that the direction of motion is in the plane of the screen. α and β are in pink and light blue, respectively. The sequence before Es and the C-termini are removed to reveal the motions of FGs more clearly. Nevertheless, the C-termini crossing Fs are important to the motions of Hs, which are still visible to a lesser extent. The helices are labeled in the still image of the movie.
(GIF)

Movie S2 **Pinch motion from a top view.** Same as Movie S1 but viewed from the opposite dimer of the stick model in foreground.
(GIF)

Movie S3 **Intradimer cooperative action.** The mechanical model of Hb dimer is put to test. A rubber band is attached to α in yellow to provide the force for closing the space between Eα and Fα, which mimics an event of ligand dissociation. This event is triggered by removing a stick that is in place to keep Eα and Fα open initially. Due to the inner workings of these helices, closing Eα and Fα causes bending of Gα, and then closing of Eβ and Fβ, and bending of Gβ. The back reaction triggered by ligand binding could also be demonstrated, which would require a compression spring to provide the force. See Fig. 3 for detail. Compare to the motion from the experimental structures in Movie S1. The experimental structures were captured either before any ligand binding or after all ligand binding events have occurred. This reverse engineered model demonstrates the motions originated from one event of ligand dissociation, which illustrates the cooperative mechanism.
(GIF)

Movie S4 **Quaternary rotation.** Same as Movie S2 except that the stick models of Cs in foreground are kept still to show the quaternary rotation.
(GIF)

Movie S5 **Cooperative quaternary rotation in action.** The mechanical model of Hb tetramer is put to test (Fig. 5). A rubber band provides the force to reduce the distance between FGs in the black dimer. While FGs in the black dimer close in, FGs in the yellow dimer also close. The black dimer rotates with respect to the yellow dimer. Compare to the motion from the experimental structures in Movie S4. The experimental structures were captured either before any ligand binding or after all ligand binding events have occurred. This reverse engineered model demonstrates the motions originated from one newly ligated dimer and transmitted to the other deoxy dimer, which illustrates the mechanism of cooperative quaternary rotation.
(GIF)
