# Supplementary figures and images for: Reverse Engineering the Cooperative Machinery of Human Hemoglobin
Source: PLoS One. 2013 Nov 27;8(11):e77363. doi: 10.1371/journal.pone.0077363 (PMC3842276; doi:10.1371/journal.pone.0077363)

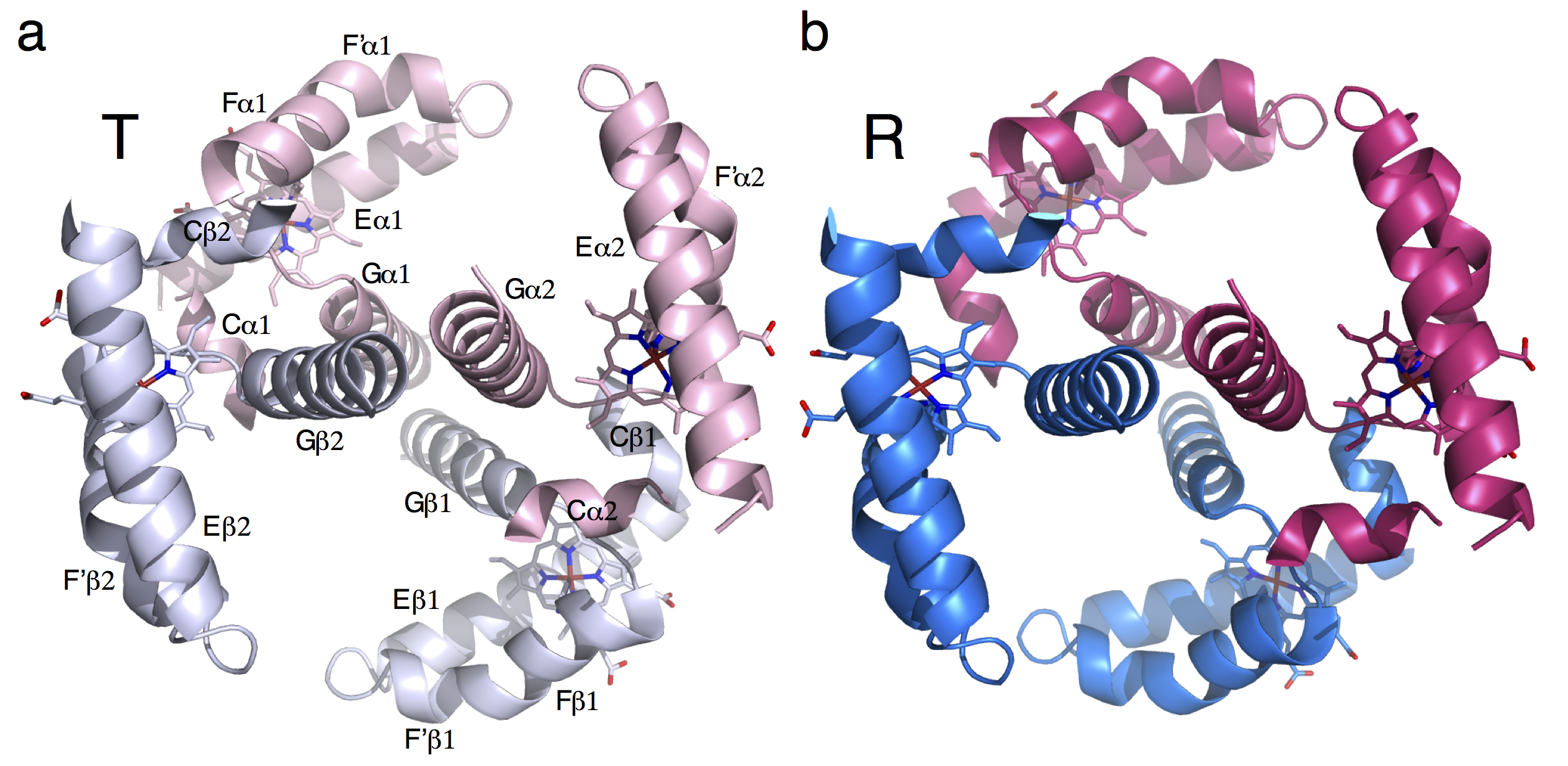

Supplement: Figure S1 — Four sides of Hb in T and R states. Four E–F pairs form four sides of Hb tetramer with the parallel, opposite sides from a same dimer. a. The four sides shape like a diamond in T state. b. They transform into a square in R state. (TIFF) [file pone.0077363.s001.tiff]

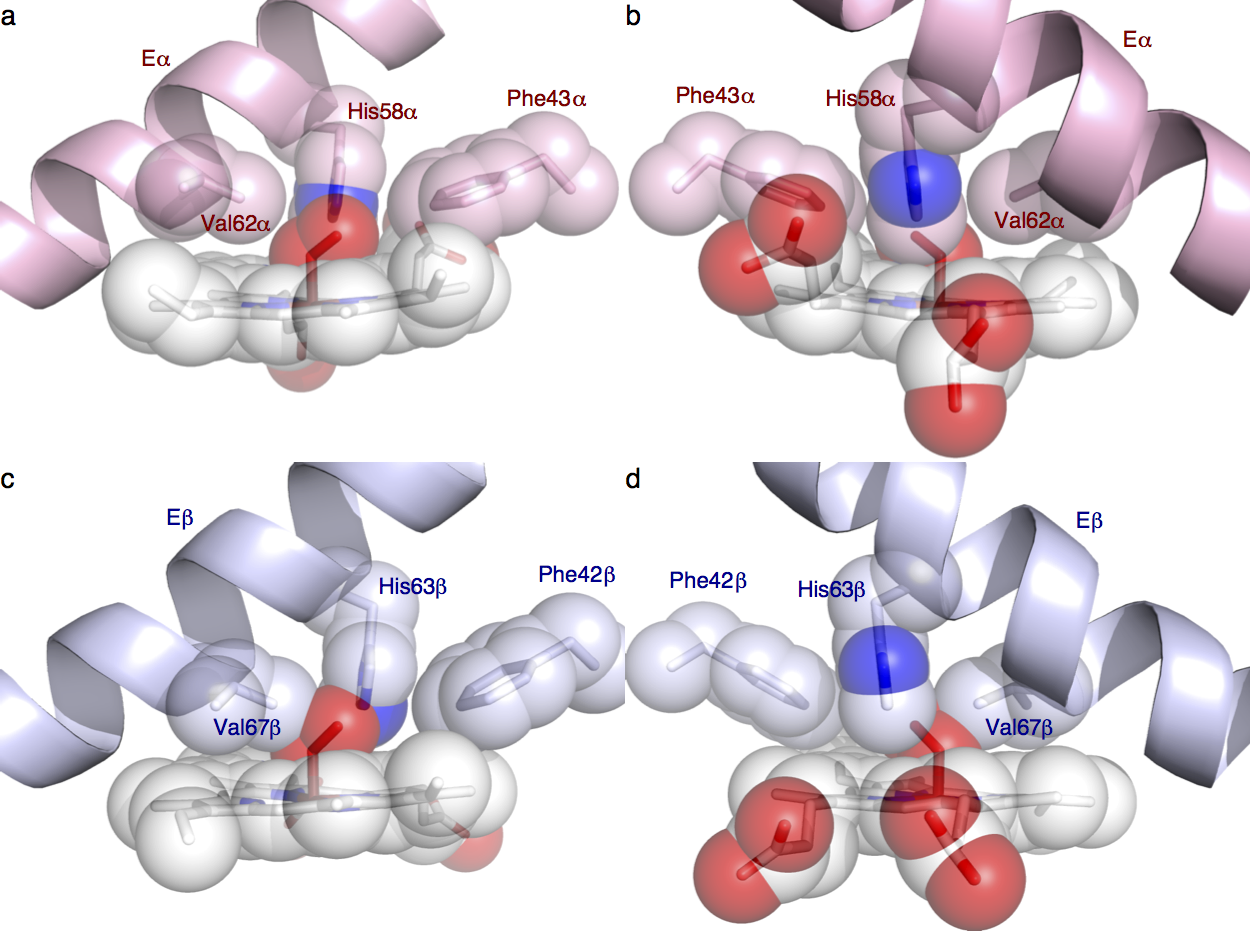

Supplement: Figure S2 — Deoxy binding site conformation. Oxygen-bound heme group in white is placed in deoxy binding site to show conflict of oxygen with distal His and Val. a and b. α in pink. c and d. β in light blue. a and c. Viewed from the interior of the molecule. b and d. Viewed from the exterior of the molecule. (TIFF) [file pone.0077363.s002.tiff]

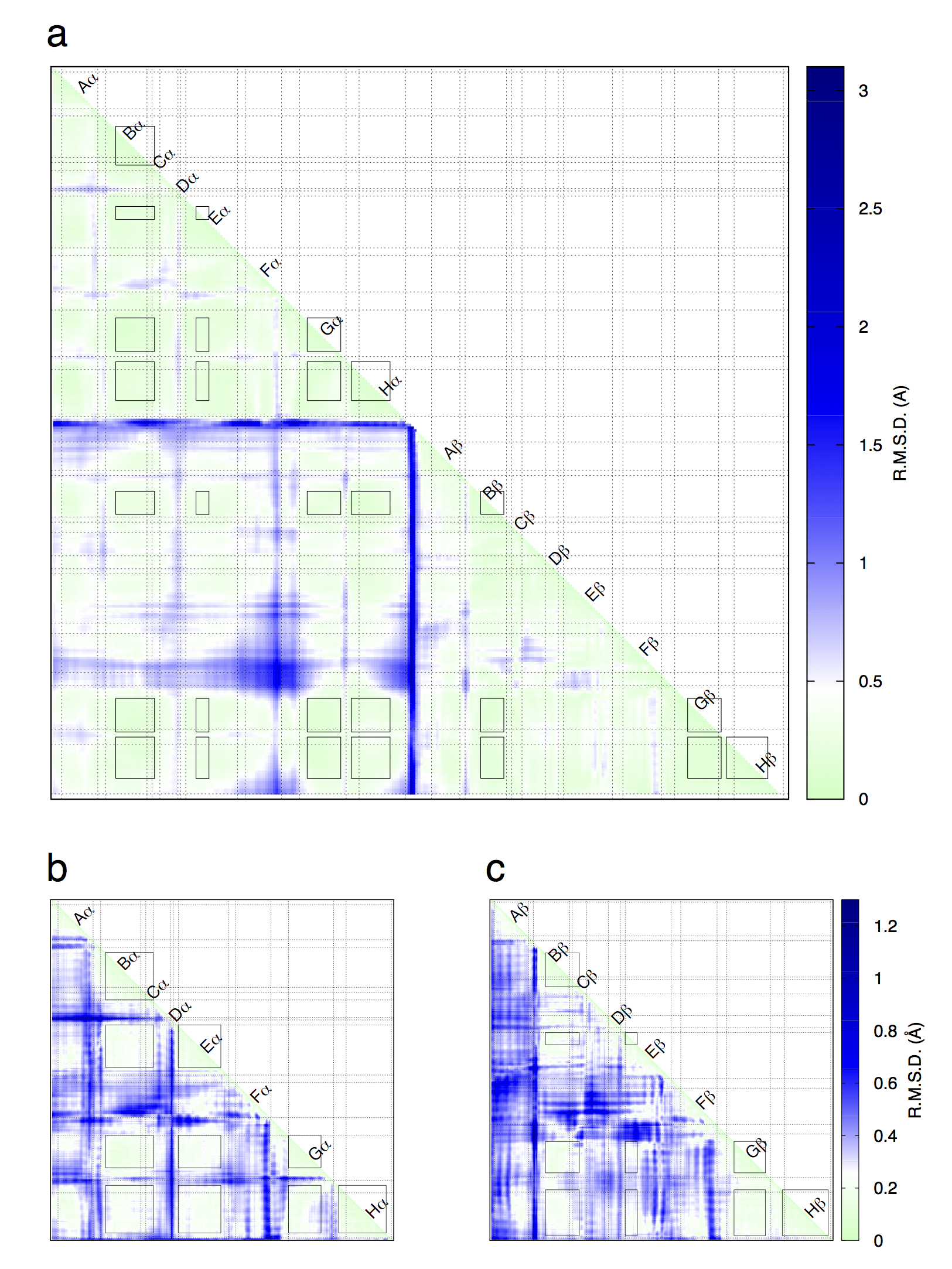

Supplement: Figure S3 — Rmsd matrices. Larger rmsd values in darker blues indicate greater structural mobility. Small values in pale green indicate invariant structural segments. Black squares on the major diagonal outline the internally rigid structural segments automatically identified. Black rectangles off the major diagonal mark the inter-segment variation. All segments must exhibit both small internal variation and small inter-segment variation to be part of the invariant structural framework. That is to say, the submatrix outlined by the black squares and rectangles must have a small average value. An automated procedure developed here evaluates the penalty upon expanding the submatrix and the saving gained by shrinking the submatrix. a. αβ. b. α. c. β. (TIFF) [file pone.0077363.s003.tiff]

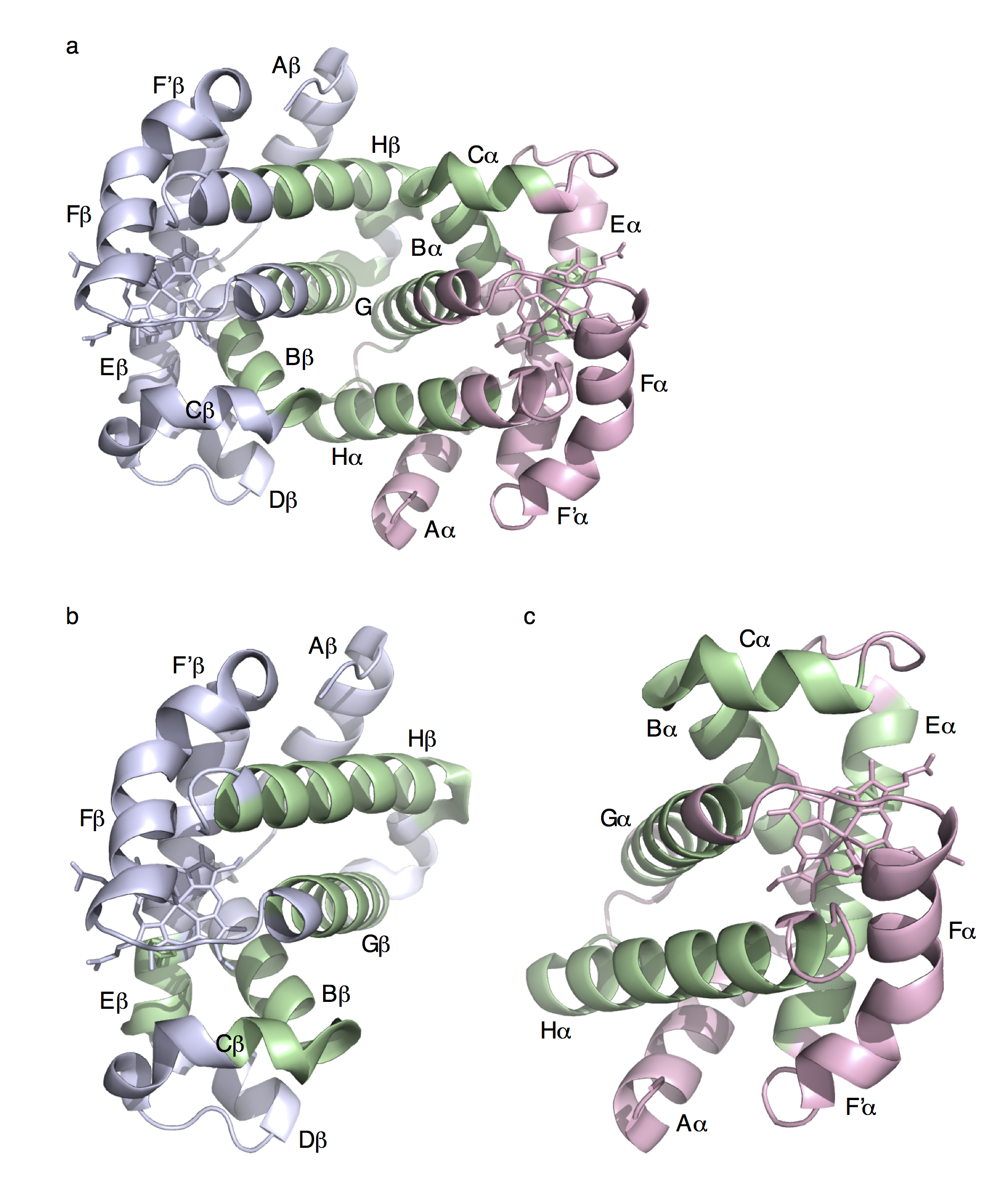

Supplement: Figure S4 — Invariant structural framework. The invariant framework identified from 560 structures is in green. The other parts of α are in pink and those of β in light blue. a. αβ. b. β. c. α. (TIFF) [file pone.0077363.s004.tiff]

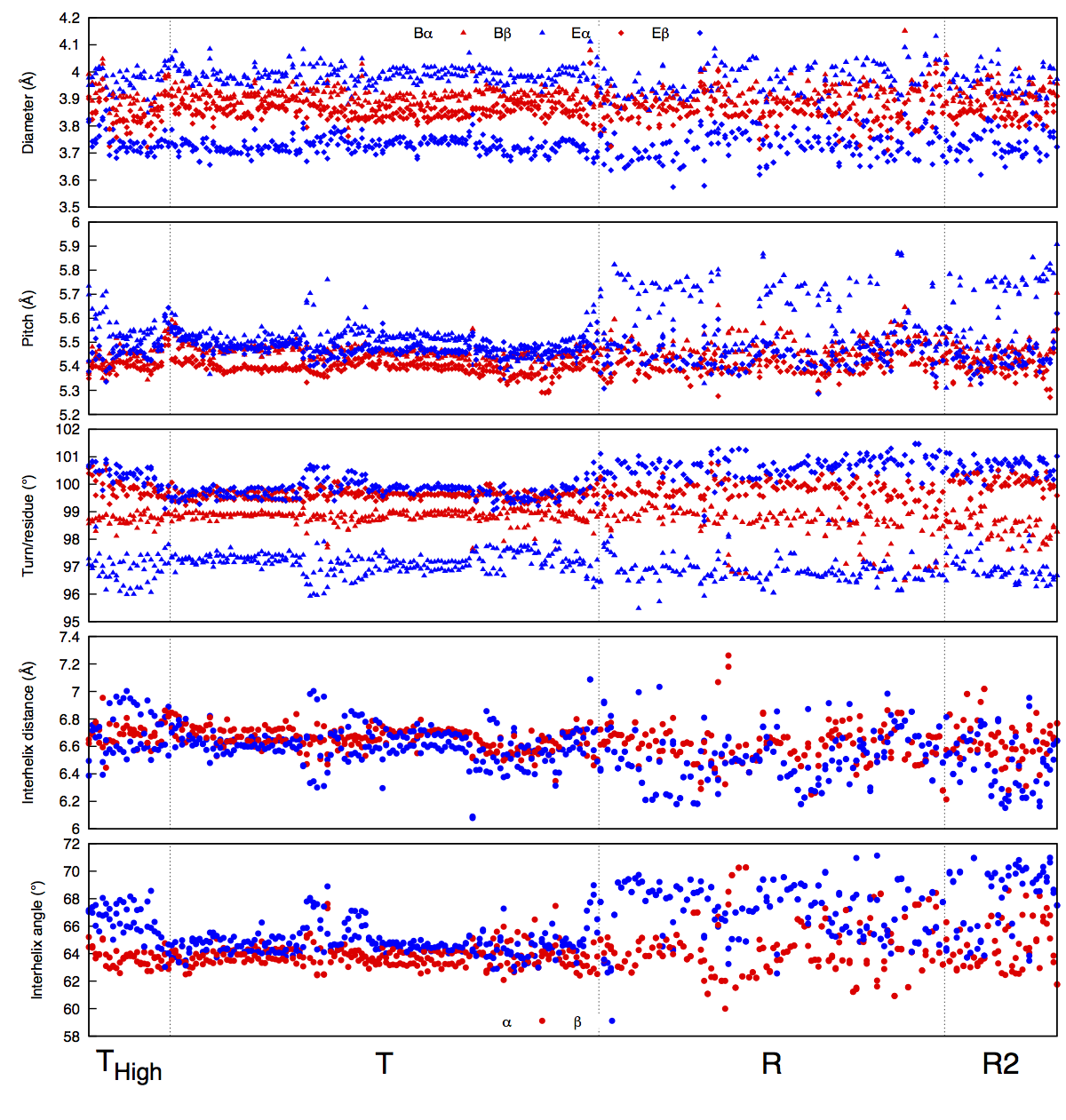

Supplement: Figure S5 — Parameterization of B and E. Helices from α and β are in red and blue, respectively. Triangle and diamond represent B and E, respectively. All helical parameters are plotted in the sequence of the reaction trajectory along THigh-T-R-R2 as identified in the companion article [5], and this applies to all similar figures below. (TIFF) [file pone.0077363.s005.tiff]

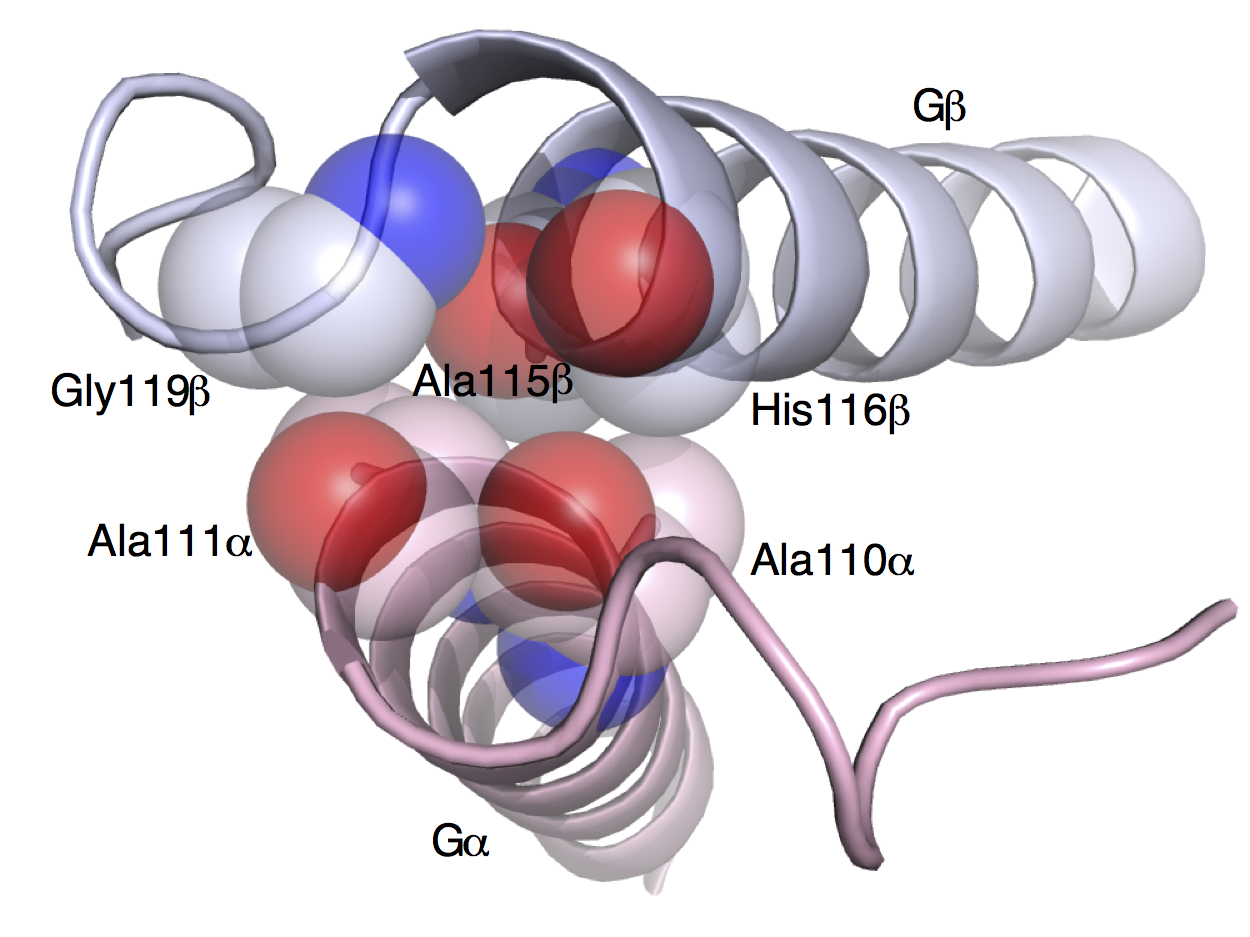

Supplement: Figure S6 — Backbone contact between Gα and Gβ. Ala111α and O are less than 3.5 Å away from Ala115βO and Gly119β , respectively. Ala110αO-His116β is slightly greater than 3.5 Å. (TIFF) [file pone.0077363.s006.tiff]

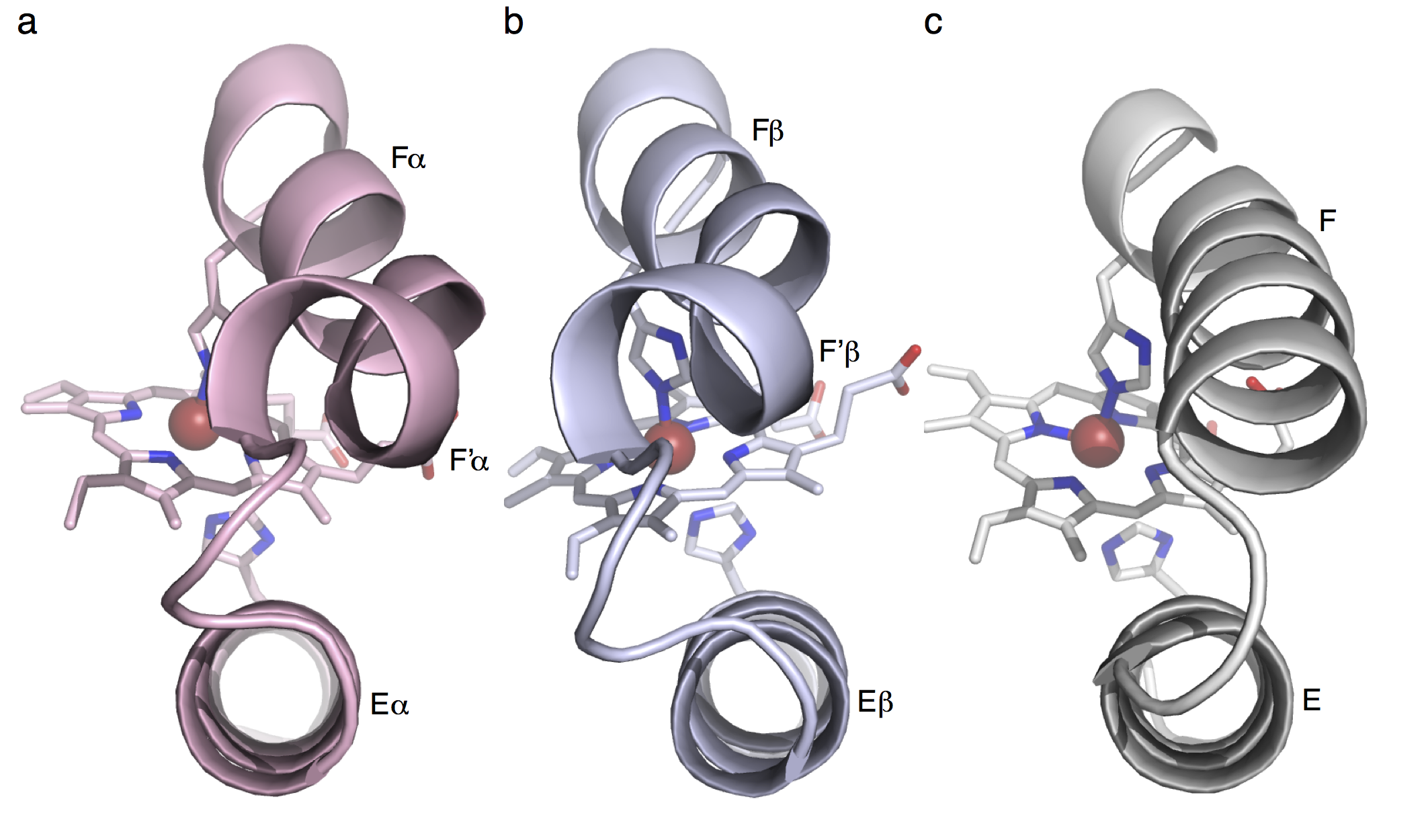

Supplement: Figure S7 — Comparison of E–F. a. Human α. b. Human β. c. Invertebrate dimeric Hb. (TIFF) [file pone.0077363.s007.tiff]

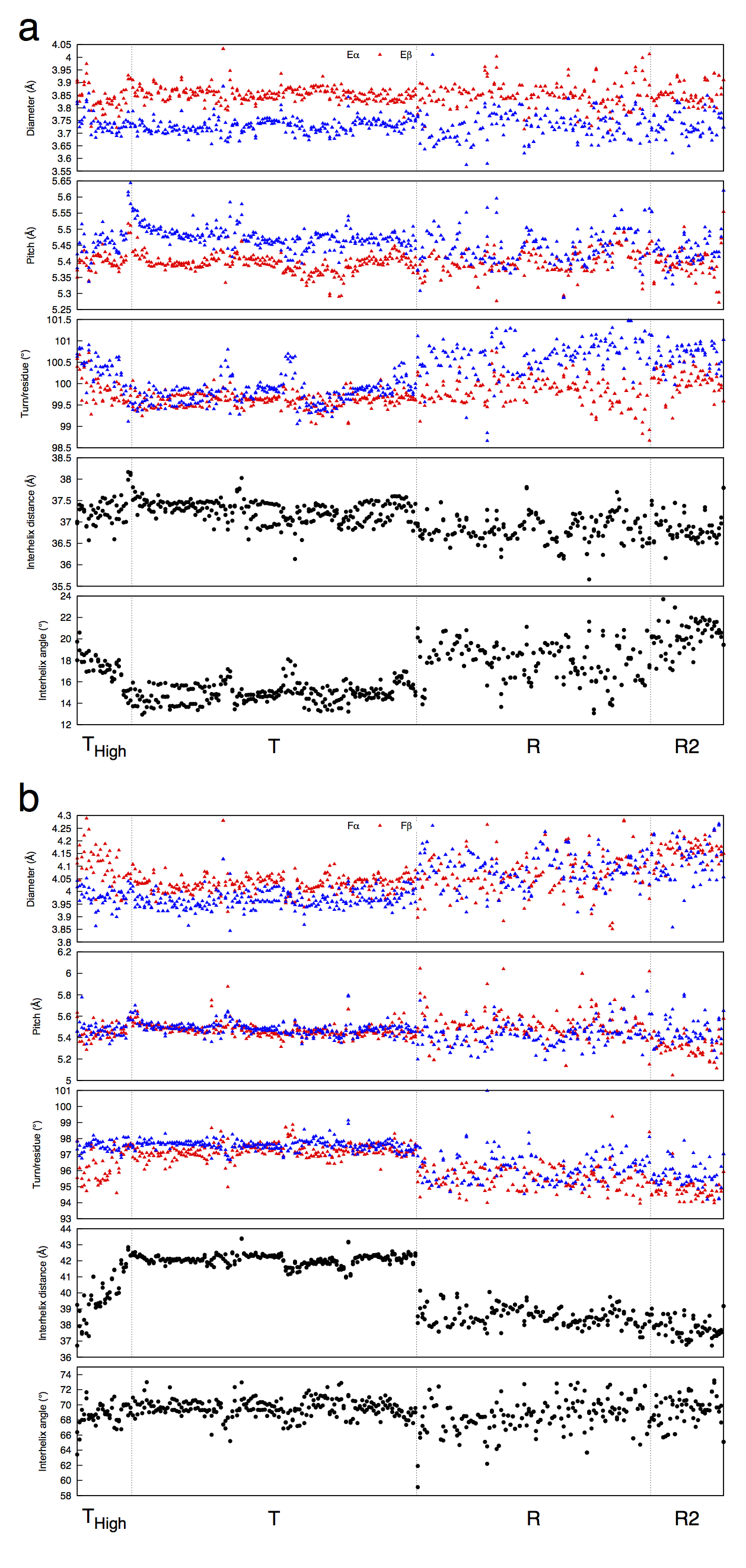

Supplement: Figure S8 — Parameterization of E (a) and F (b). Helices from α and β are in red and blue, respectively. Interhelix parameters between α and β are in black. See also Fig. S5 legend. (TIFF) [file pone.0077363.s008.tiff]

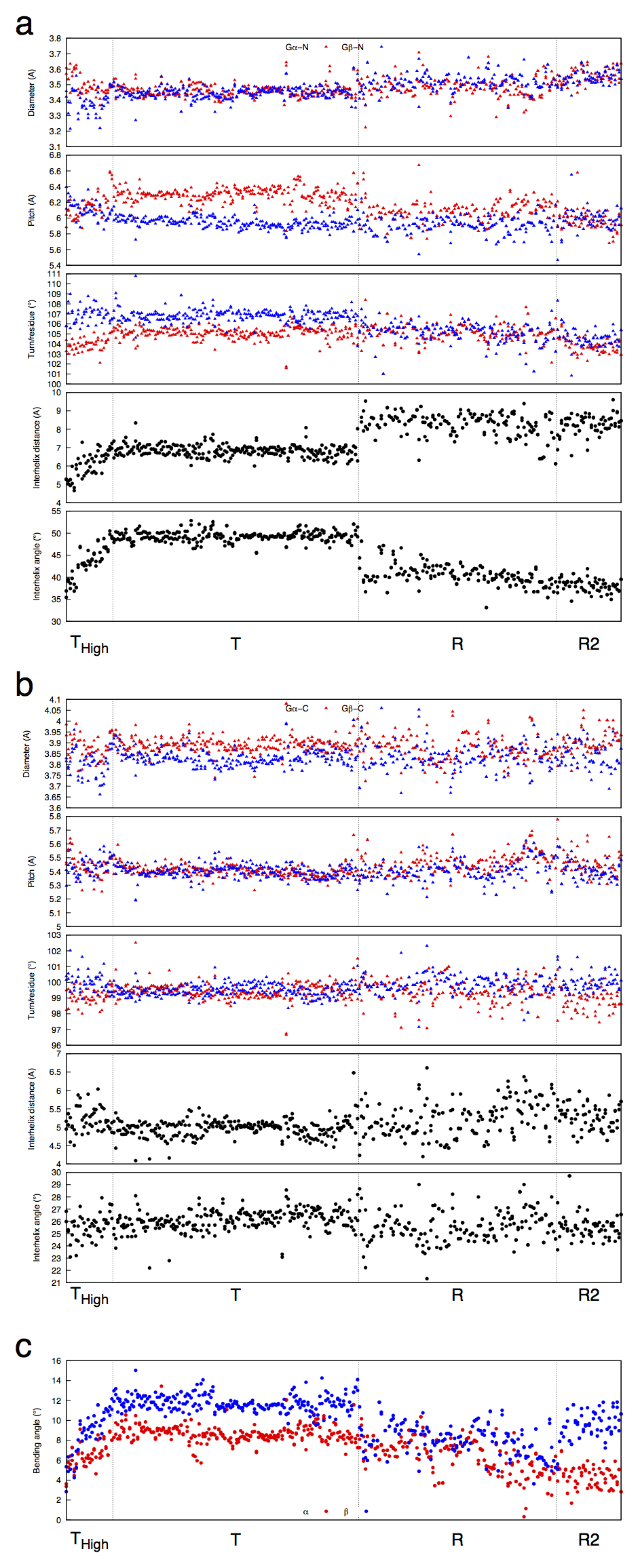

Supplement: Figure S9 — Parameterization of G. Helices from α and β are in red and blue, respectively. Interhelix parameters between α and β are in black. a. N-terminal section of G in 3/10 conformation. b. C-terminal section of G in α conformation. c. Bending angle between N- and C-terminal sections. Notice that the 3/10 helices have significantly smaller diameter, but larger pitch and turn per residue compared to α helices. See also Fig. S5 legend. (TIFF) [file pone.0077363.s009.tiff]

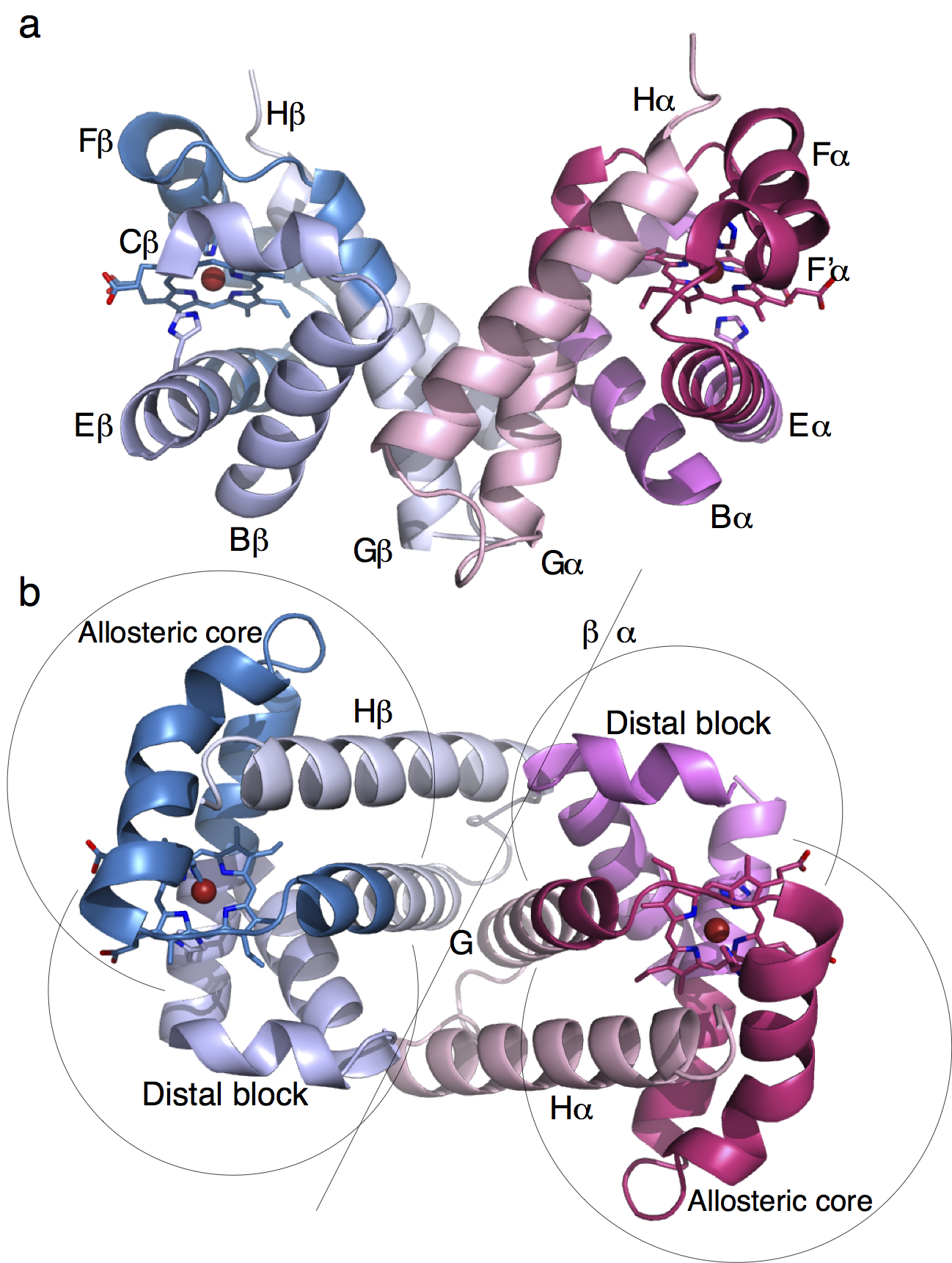

Supplement: Figure S10 — Allosteric core and distal block. α and β are in warm and cool colors, respectively. The allosteric cores and distal blocks are in darker and lighter colors. a. Side view with dimer interface facing up. b. Top view directly into the dimer interface from the opposite dimer. (TIFF) [file pone.0077363.s010.tiff]

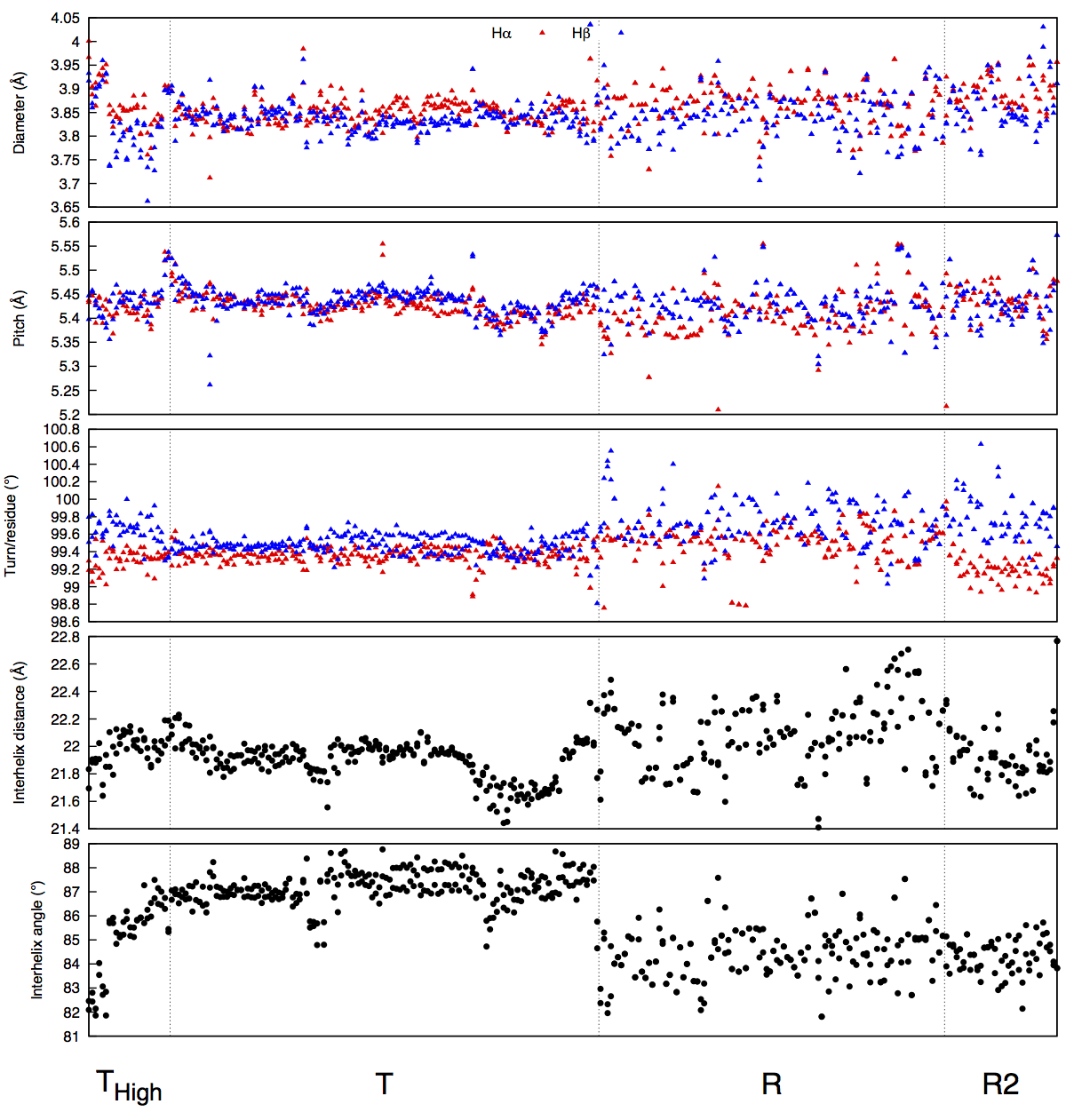

Supplement: Figure S11 — Parameterization of H. α and β are in red and blue, respectively. Interhelix parameters are in black. See also Fig. S5 legend. (TIFF) [file pone.0077363.s011.tiff]

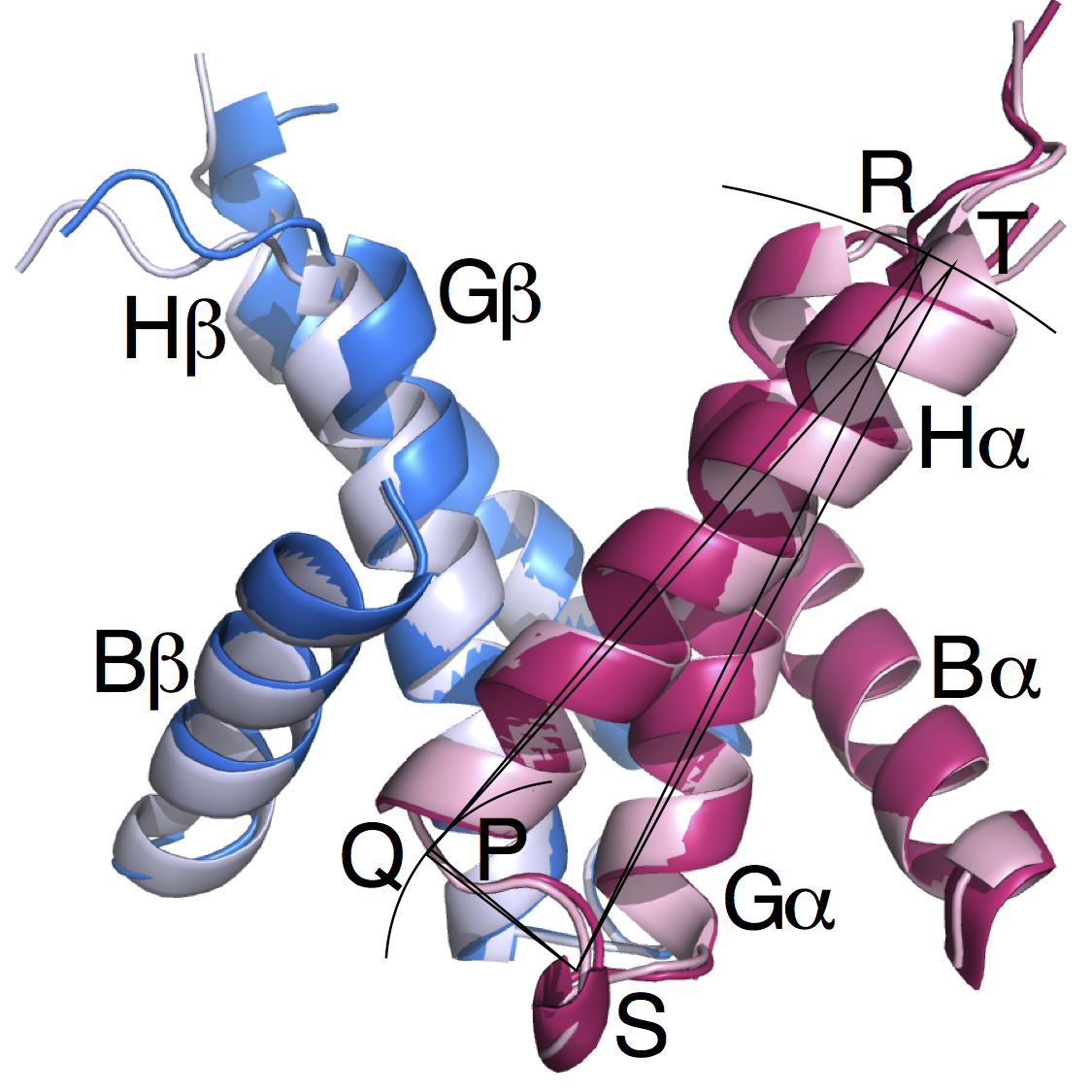

Supplement: Figure S12 — Demagnification of motion transmission. α and β are in warm and cool colors, respectively. Deoxy and ligated states are in light and dark colors. (TIFF) [file pone.0077363.s012.tiff]

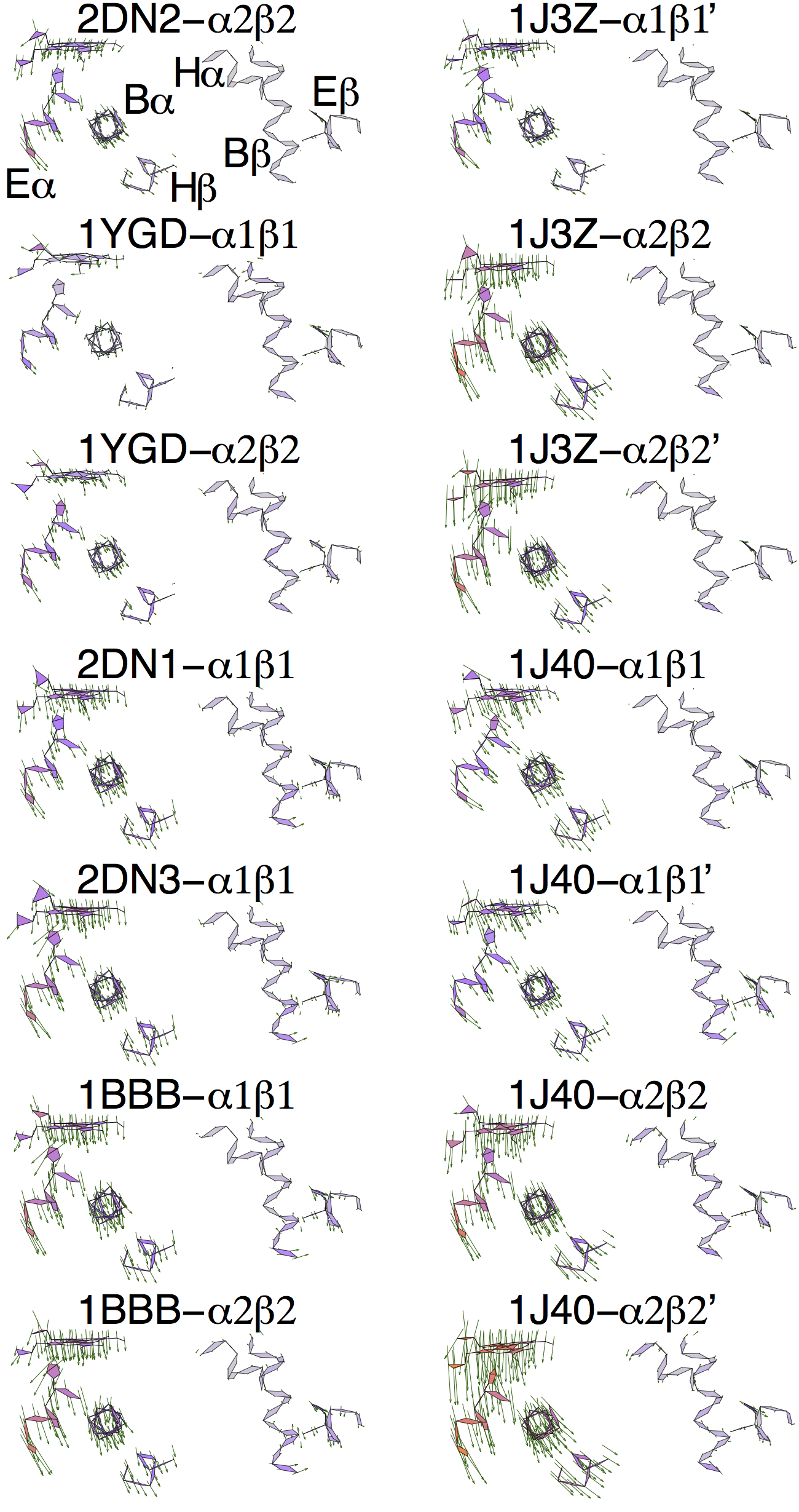

Supplement: Figure S13 — Relative motion between Bα-Eα-Hβ and Bβ-Eβ-Hα. Each panel is labeled by the source of a dimer structure. Helices are labeled on the first panel only. All structures are aligned to 2DN2-α1β1 by least-square fitting of Bβ-Eβ-Hα on the right side of each panel. Atomic displacements of Bα-Eα-Hβ on the left side of each panel are marked by green arrows. Each arrow is three times as long as the real displacement. (TIFF) [file pone.0077363.s013.tiff]

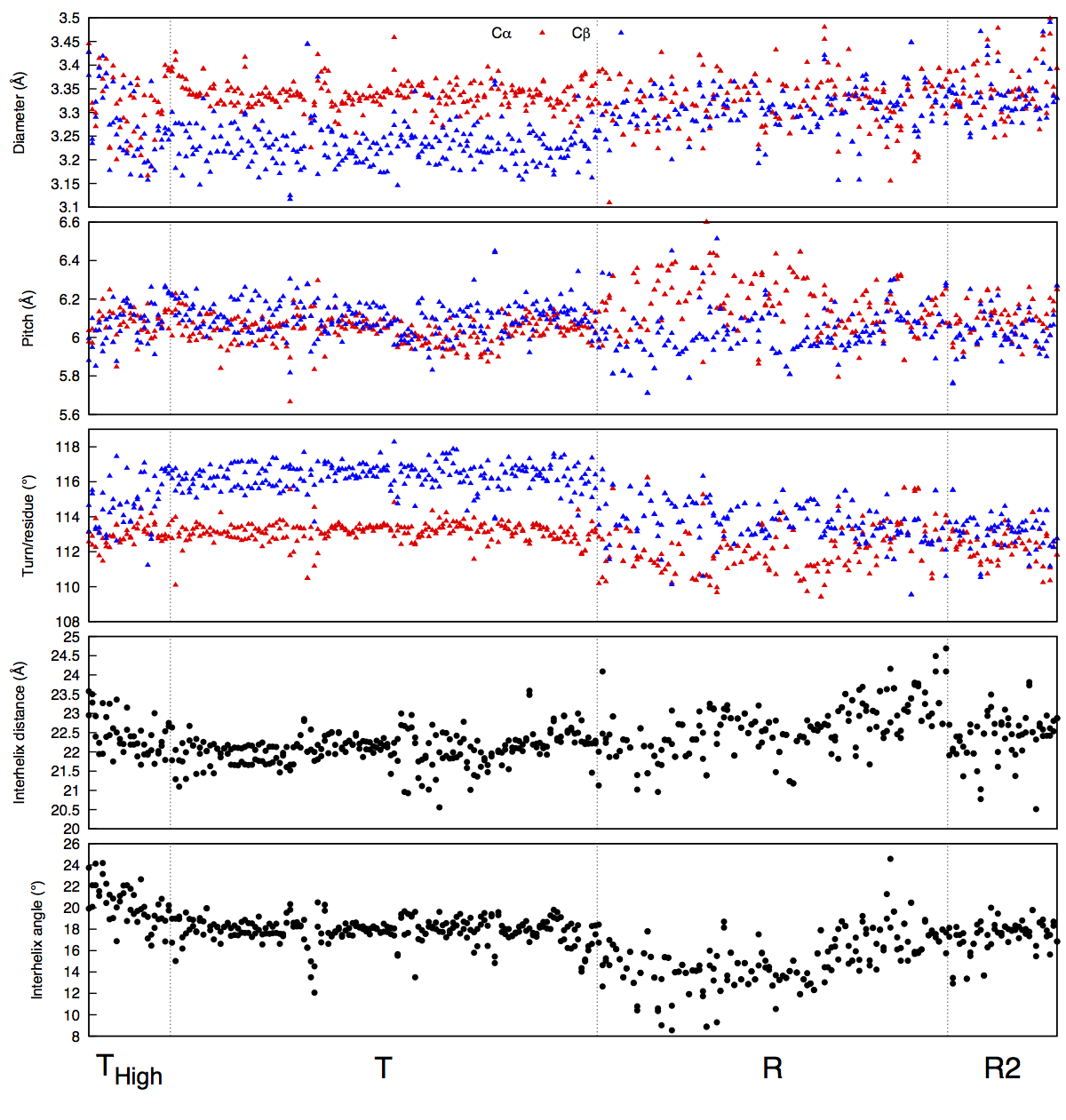

Supplement: Figure S14 — Parameterization of C. α and β are in red and blue, respectively. Interhelix parameters are in black. See also Fig. S5 legend. (TIFF) [file pone.0077363.s014.tiff]

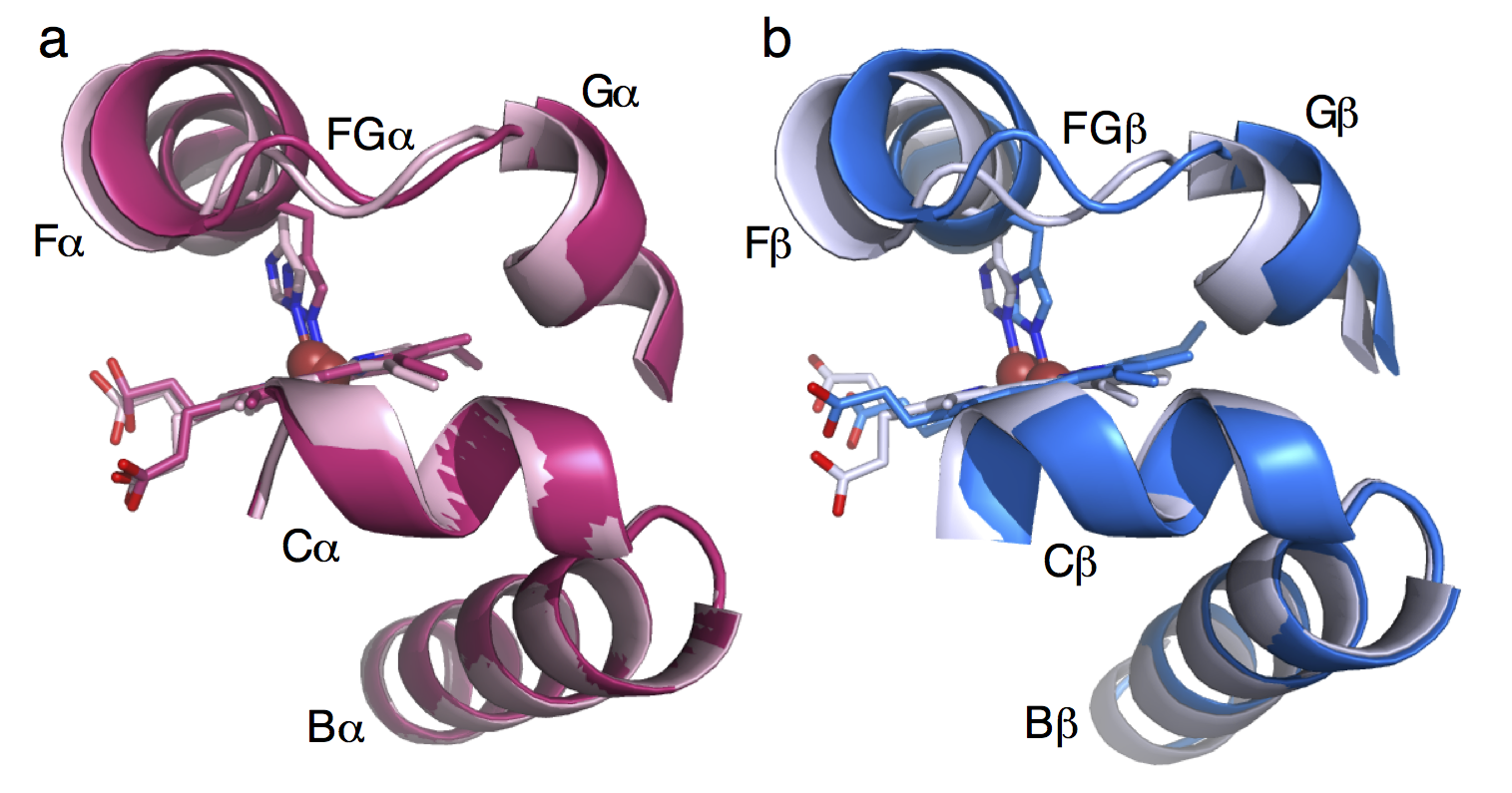

Supplement: Figure S15 — Small interdimer couplings Cs and FGs. C and FG in a same subunit run antiparallel to each other and both are roughly parallel to the heme plane. Deoxy and ligated structures are in light and dark colors, respectively. a. α. b. β. (TIFF) [file pone.0077363.s015.tiff]

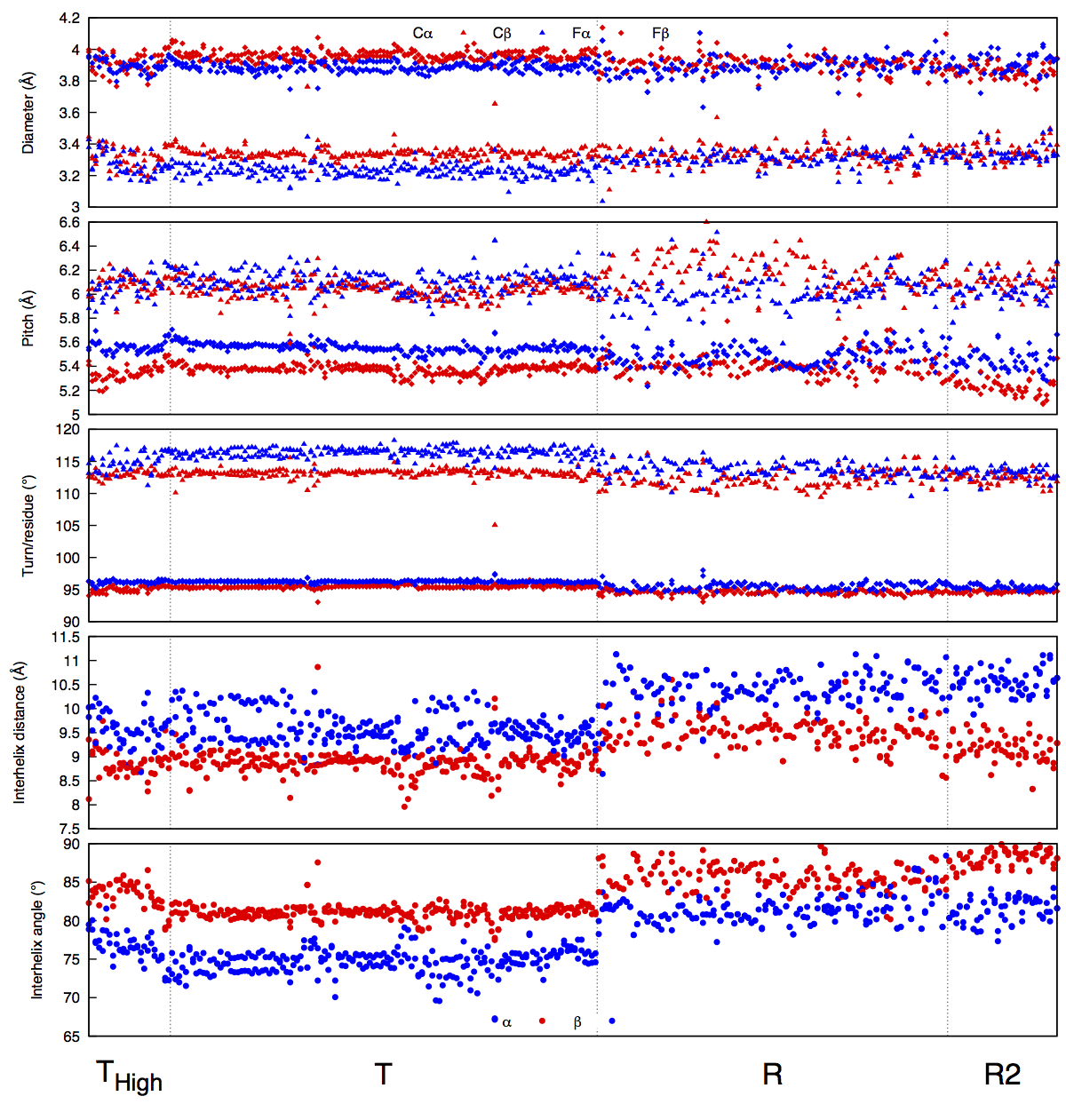

Supplement: Figure S16 — Parameterization of C and F. Helices from α and β are in red and blue, respectively. See also Fig. S5 legend. (TIFF) [file pone.0077363.s016.tiff]

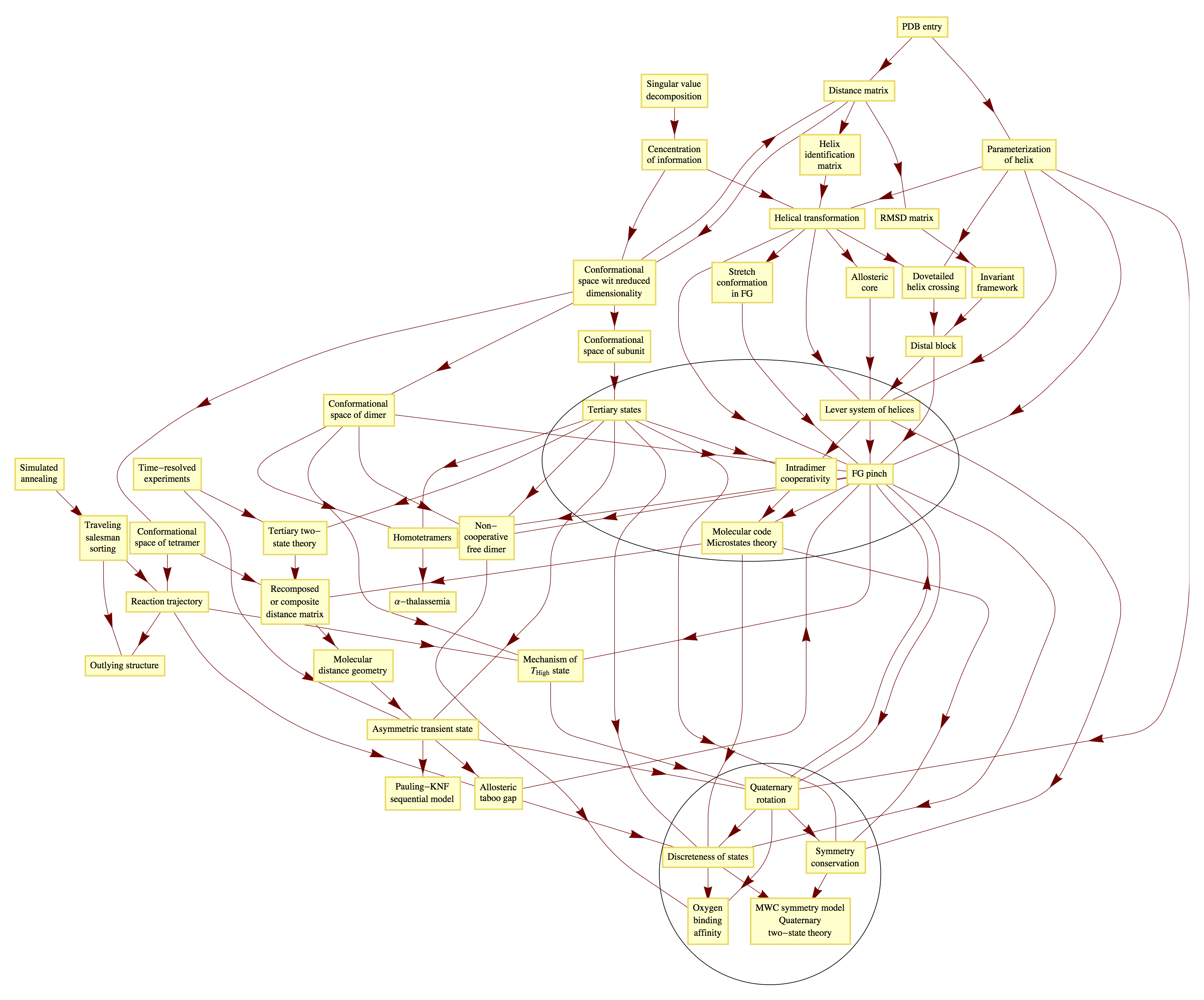

Supplement: Figure S17 — A directed graph of the topics in the companion articles. The main topics presented in these companion articles [5] are linked by the directed edges that indicate the direction of reasoning. The overall conclusion from these two articles is the molecular mechanism of a cluster of nodes in the circle. The oval at the center of the graph outlines the key findings that play a central role in the analysis. (TIFF) [file pone.0077363.s017.tiff]

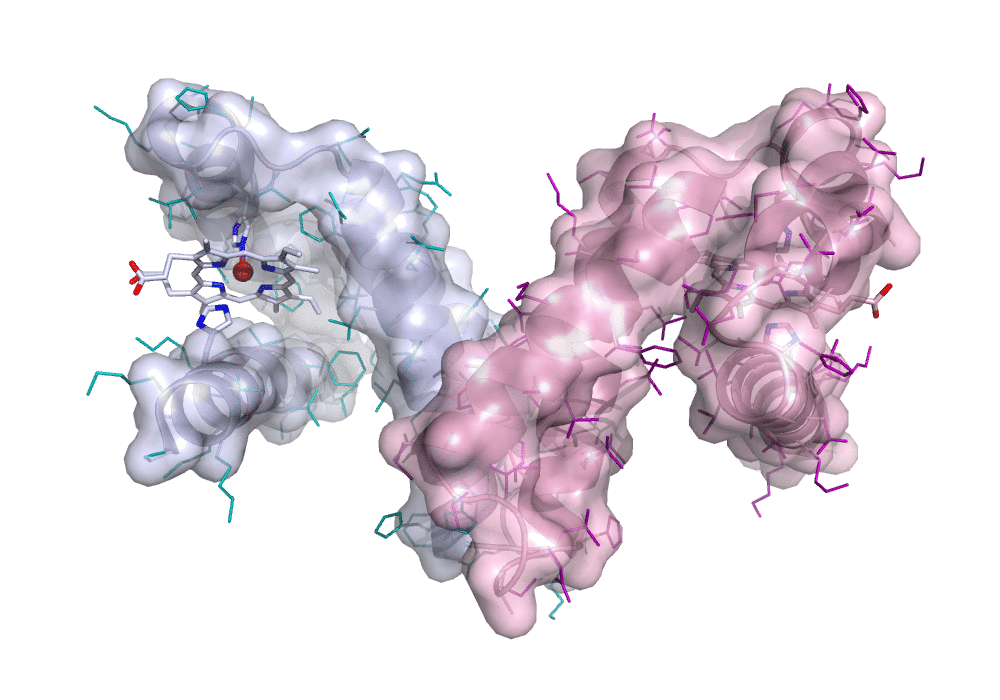

Supplement: Movie S1 — Pinch motion from a side view. The pinch motion of αβ is viewed from a sideway so that the direction of motion is in the plane of the screen. α and β are in pink and light blue, respectively. The sequence before Es and the C-termini are removed to reveal the motions of FGs more clearly. Nevertheless, the C-termini crossing Fs are important to the motions of Hs, which are still visible to a lesser extent. The helices are labeled in the still image of the movie. (GIF) [file pone.0077363.s018.gif]

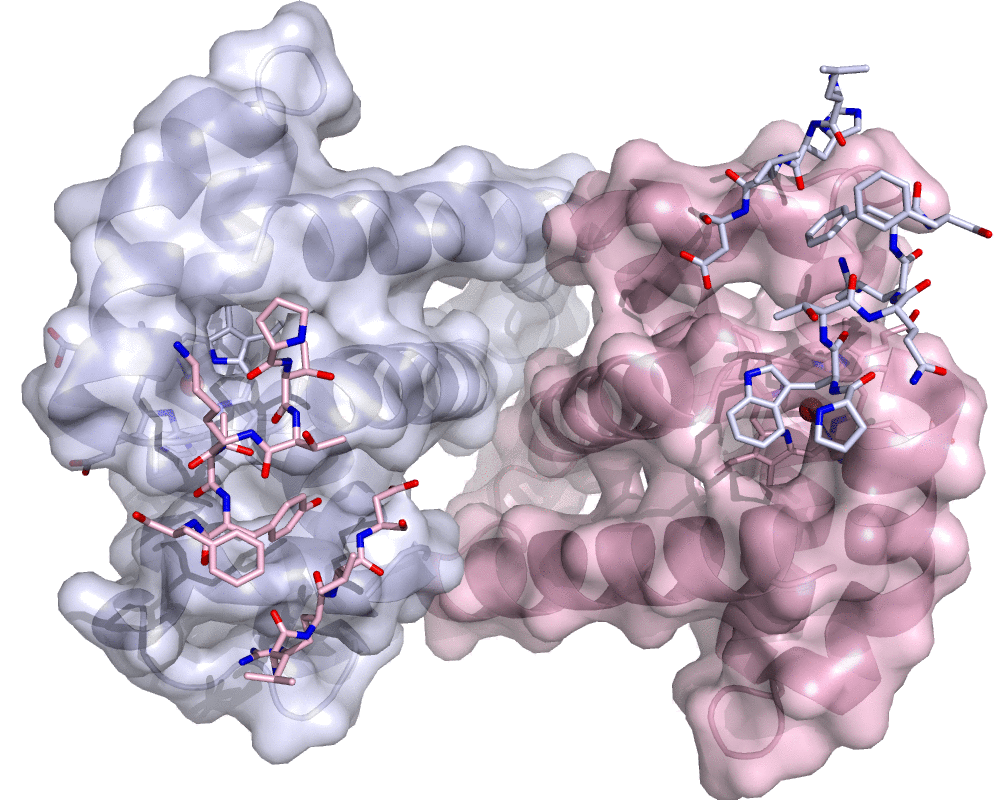

Supplement: Movie S2 — Pinch motion from a top view. Same as Movie S1 but viewed from the opposite dimer of the stick model in foreground. (GIF) [file pone.0077363.s019.gif]

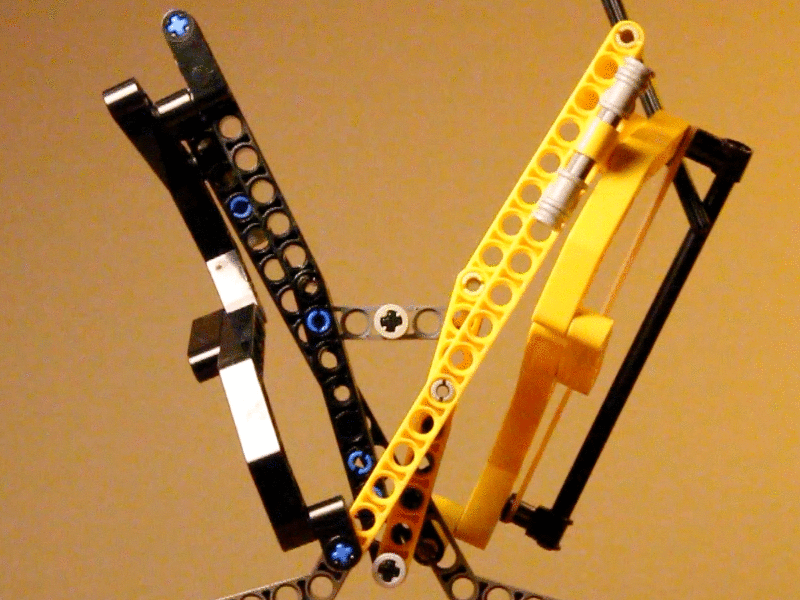

Supplement: Movie S3 — Intradimer cooperative action. The mechanical model of Hb dimer is put to test. A rubber band is attached to α in yellow to provide the force for closing the space between Eα and Fα, which mimics an event of ligand dissociation. This event is triggered by removing a stick that is in place to keep Eα and Fα open initially. Due to the inner workings of these helices, closing Eα and Fα causes bending of Gα, and then closing of Eβ and Fβ, and bending of Gβ. The back reaction triggered by ligand binding could also be demonstrated, which would require a compression spring to provide the force. See Fig. 3 for detail. Compare to the motion from the experimental structures in Movie S1. The experimental structures were captured either before any ligand binding or after all ligand binding events have occurred. This reverse engineered model demonstrates the motions originated from one event of ligand dissociation, which illustrates the cooperative mechanism. (GIF) [file pone.0077363.s020.gif]

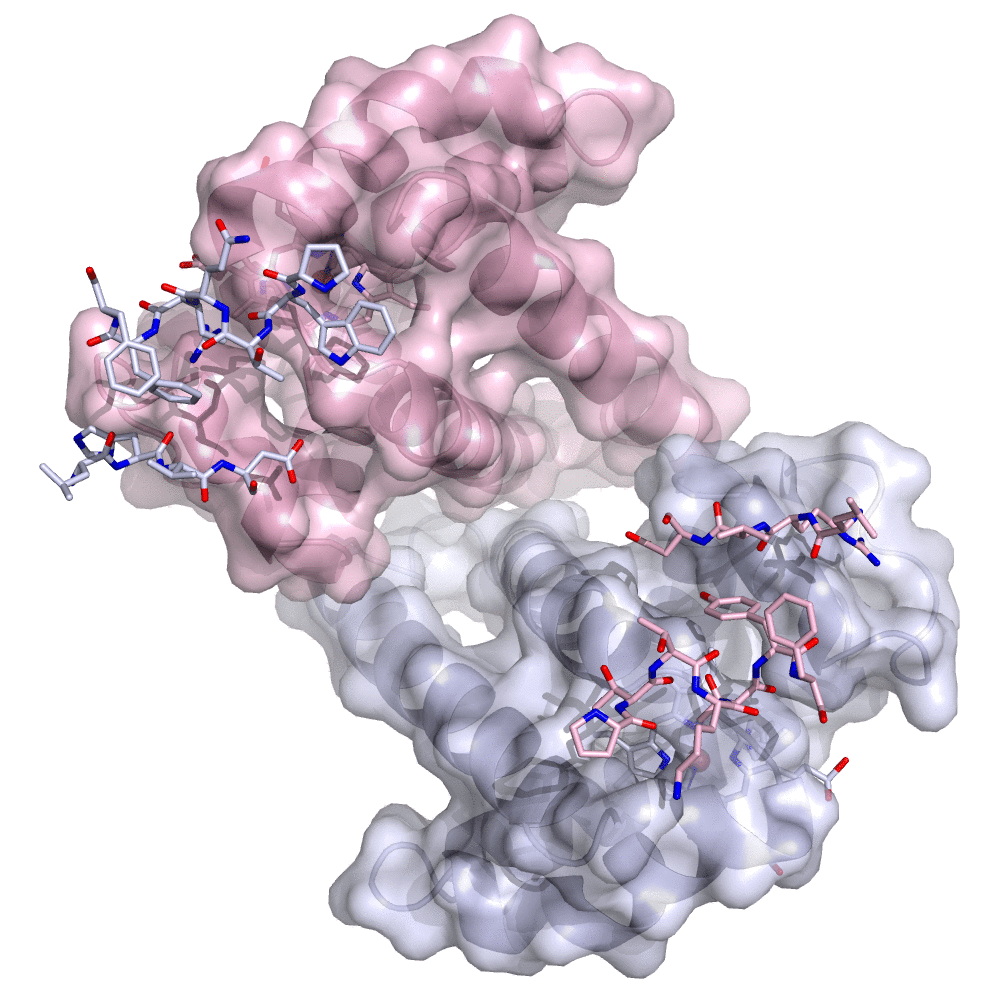

Supplement: Movie S4 — Quaternary rotation. Same as Movie S2 except that the stick models of Cs in foreground are kept still to show the quaternary rotation. (GIF) [file pone.0077363.s021.gif]

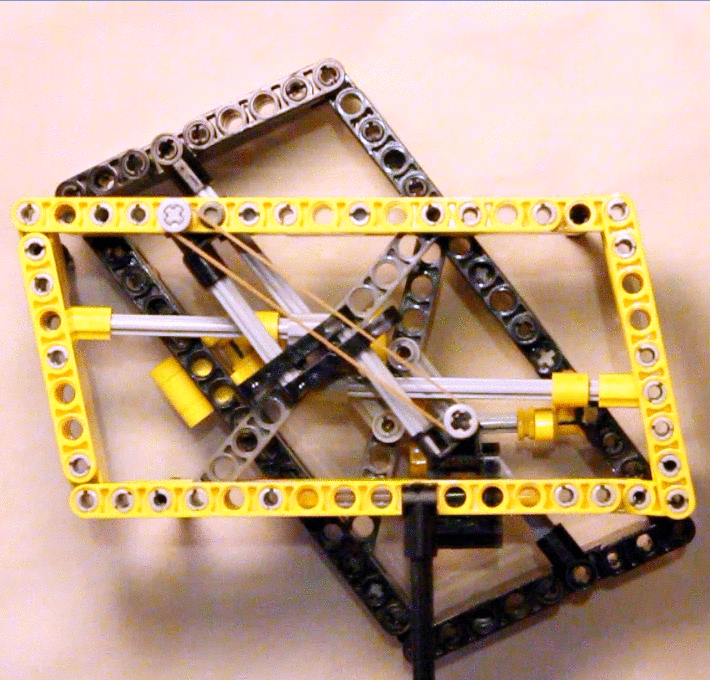

Supplement: Movie S5 — Cooperative quaternary rotation in action. The mechanical model of Hb tetramer is put to test (Fig. 5). A rubber band provides the force to reduce the distance between FGs in the black dimer. While FGs in the black dimer close in, FGs in the yellow dimer also close. The black dimer rotates with respect to the yellow dimer. Compare to the motion from the experimental structures in Movie S4. The experimental structures were captured either before any ligand binding or after all ligand binding events have occurred. This reverse engineered model demonstrates the motions originated from one newly ligated dimer and transmitted to the other deoxy dimer, which illustrates the mechanism of cooperative quaternary rotation. (GIF) [file pone.0077363.s022.gif]
